# Unravelling the synergistic interaction of *Thrips tabaci* and newly recorded, *Thrips parvispinus* with *Alternaria porri* (Ellis.) Cif., inciting onion purple blotch

**DOI:** 10.3389/fmicb.2024.1321921

**Published:** 2024-03-05

**Authors:** Shubham Saini, Kushal Raj, Anil Kumar Saini, Rakesh Kumar, Ankit Saini, Aslam Khan, Pankaj Kumar, Geeta Devi, Mukul Kumar Bhambhu, Cindy L. McKenzie, Makhan Lal, Leela Wati

**Affiliations:** ^1^Department of Plant Pathology, CCS Haryana Agricultural University, Hisar, Haryana, India; ^2^Department of Vegetable Science, CCS Haryana Agricultural University, Hisar, Haryana, India; ^3^Department of Entomology, CCS Haryana Agricultural University, Hisar, Haryana, India; ^4^Department of Nematology, CCS Haryana Agricultural University, Hisar, Haryana, India; ^5^ARS Horticultural Research Laboratory, USDA, Fort Pierce, FL, United States; ^6^Department of Microbiology, CCS Haryana Agricultural University, Hisar, Haryana, India

**Keywords:** *Alternaria porri*, injury, thrips, *Thrips tabaci*, *Thrips parvispinus*, onion, purple blotch

## Abstract

Onion purple blotch is the most indispensable foliar disease of crop and has become a major concern for farmers and research fraternity. An attempt to investigate the role of injury in parasitism by *Alternaria porri* indicated that disease incidence and severity enhance considerably with injury. Thrips injured plants inoculated with *A. porri* presented 100% incidence and 52–72% severity while mechanically injured plants inoculated with *A. porri* showed 60–70% incidence and 28–34% severity. The uninjured plants showed considerably less disease incidence (30–40%) and severity (10–16%). Injured inoculated plants presented reduced leaf length and leaf area while the leaf diameter remained unaffected. The lesion number, lesion length and size was substantially enhanced with concomitant infestation of pest and pathogen. *Thrips tabaci* injury led to more pronounced symptoms of purple blotch compared to *Thrips parvispinus* injury. There was substantial decrease in photosynthetic rate and chlorophyll content with stress imposed on plant whilst the relative stress injury was enhanced. The induction of injury and inoculation of *A. porri* had an impact on the concentration of total phenolics, total soluble sugars, total proteins and hydrogen peroxide in onion leaves. *A. porri* combined with injury caused a more pronounced decrease in total soluble sugars and total protein content while enhancement in total phenolics and hydrogen peroxide content compared to uninjured plants. The dynamic nature of morpho-physiological and biochemical changes owing to stress conditions imposed on onion plant adds an extra layer of complexity in understanding the onion plant physiology and their ability to work out in response to challenging environment conditions.

## Introduction

Thrips present a substantial threat to global agricultural and horticultural crop production, as their association with plant diseases goes beyond their role as herbivores (Ullman et al., 1997). While the relationship between thrips and plant virus transmission is well-documented, with examples such as *Frankliniella occidentalis* transmitting tomato spotted wilt virus and *Thrips tabaci* vectoring iris yellow spot virus (Wijkamp et al., 1995; Kritzman et al., 2001), their interaction with fungal pathogens has not been thoroughly explored to date. Several thrips species known for transmitting tospoviruses have also been identified as carriers of fungal and bacterial plant pathogens (Whitfield et al., 2005; Diaz-Montano et al., 2011). Despite this, the depth of understanding regarding the interaction of thrips with fungal pathogens has been limited. Examples such as the association of flower thrips (*Frankliniella* sp.) with Fusarium ear rot (*Fusarium verticillioides*) in maize (Farrar and Davis, 1991) and their involvement in the occurrence of hardlock (*Fusarium verticillioides*) in cotton (Mailhot et al., 2007) underscore the need for a comprehensive exploration of thrips interactions with various plant pathogens beyond viral transmission.

Onion thrips, *Thrips tabaci* Lindeman (Thysanoptera: Thripidae) present a significant to agricultural crops globally (Lewis, 1997), whose presence recorded in 120 countries (CAB International, 2021). They have wide host range (Lewis, 1997), however, onion is its preferred host (Gill et al., 2015). The highly invasive nature of pest may be attributed to its small size, cryptic behavior, polyphagy, and short generation time, parthenogenetic mode of reproduction, high reproductive potential and dispersal ability (North and Shelton, 1986; Morse and Hoddle, 2006; Diaz-Montano et al., 2011). They possess asymmetrical mouth parts, comprising a singular mandibular stylet that is used to pierce plant tissue while feeding (Chisholm and Lewis, 1984), thereby damaging the epidermal tissues and mesophyll cell (Huckabn and Coble, 1991). The loss of integrity of host epidermal walls paves the way for entry of plant pathogens (Cartwright et al., 1995; Childers and Achor, 1995), thereby acting as a source of secondary infection by fungi and bacteria (McKenzie et al., 1993). A positive correlation of onion thrips population and pathogen infection has been indicated by many workers (Yarwood, 1943; Dutta et al., 2014). Onion thrips are known to mechanically vector Stemphylium leaf blight (Leach et al., 2020a,b) and bacterial leaf blight (Grode et al., 2017, 2019). Recently the infestation of South East Asian thrips, *Thrips parvispinus* was observed on onion crop (Saini et al., 2022), which may pose a significant threat to crop in near future.

Onion thrips are often found in field concurrently with fungal pathogens such as *Colletotrichum coccodes* (Waller) S. Hughes (Glomerellales: Glomerellaceae), inciting anthracnose disease (Rodriguez-Salamanca et al., 2012); *Stemphylium vesicarium* (Wallr.) E.G. Simmons, the causal agent of leaf blight (Leach et al., 2020a,b); *Alternaria porri* (Ellis.) Cif. causing purple blotch (Saini et al., 2022). Disease incidence and severity had been positively correlated with feeding damage and thrips population, indicating contribution of thrips in pathogen infection. Amongst these, purple blotch is one of the most destructive foliar diseases of crop, prevalent in all onion-growing regions of the world (Kareem et al., 2012). The disease led to heavy losses in bulb as well as seed yield, which may reach to extent of 85% under severe conditions (Veeraghanti et al., 2017). The fungus is known to incite quiescent infection and enters the tissue where it remains dormant till the favorable conditions are met. The pathogen develops dark brown to purplish necrotic lesions on leaf tissue with peculiar zonation of dark and light concentric rings giving target board appearance to the lesion (Saini and Raj, 2022).

*A. porri* is able to penetrate plant directly through epidermal cells as well as through stomata; however it also appears to use areas of insect damage as an alternative penetration site. Thus, the feeding wounds caused by thrips may enhance and facilitate the entry and development of *A. porri*. Infection of *A. porri* on onion leaves led to necrotic lesions, with consequent reduction in yield (Miller, 1983) and similar effects by thrips feeding on crop were noticed by Edelson et al. (1989). The impact of other key pests was kept constant by both the researchers, thereby exploring singular effects of the two key pests of the crop. The pest, however, rarely occurs alone, yet little information is available concerning the concomitant occurrence of *A. porri* and thrips. Building upon the insights provide by McKenzie et al. (1993) regarding the pivotal role of thrips induced feeding damage in proliferation of *A. porri* and the subsequent susceptibility of crop to pathogen infection, the present study investigates the extent to which thrips-induced feeding damage (*Thrips tabaci* and *Thrips parvispinus*) influences the proliferation of *A. porri* and the subsequent morpho-physio and biochemical changes in onion. The inclusion of Thrips parvispinus as a newly recorded species suggests a potential gap in knowledge regarding the specific interactions between different thrips species and their impact on onion purple blotch. By addressing these objectives, the study aims to contribute valuable knowledge to the field, bridging gaps in our understanding of the complex interactions between thrips feeding damage and the subsequent impact on *A. porri* infection in onion crops.

## Materials and methods

### Preparation of pathogen inoculum

The four isolates of *A. porri* from the previous study by Saini and Raj (2022) were used for experimentation purpose. The isolates were designated as Ap 1 (Hisar isolate), Ap 2 (Mewat isolate), Ap 3 (Ambala isolate) and Ap 4 (Panchkula isolate), characterized at molecular level using ITS-rRNA primers (White et al., 1990) and the resulting sequences were submitted to National Center for Biotechnology Information (NCBI) for obtaining accession number. Single conidium-derived isolates grown on Potato Carrot Agar in petri plates were flooded with 15 ml of sterile water and a surfactant (1 drop Tween 20/100 ml water), conidia were scraped into suspension with a spatula and stirred on a magnetic stirrer for 20 min to detach conidia from conidiophores and sieved through a 150 μm sieve to remove large pieces of mycelium. The resulting conidial suspension was counted using a haemocytometer and adjusted to mean conidial count of 1 × 10^6^ per ml.

### Collection and identification of thrips

**Figure 1:**
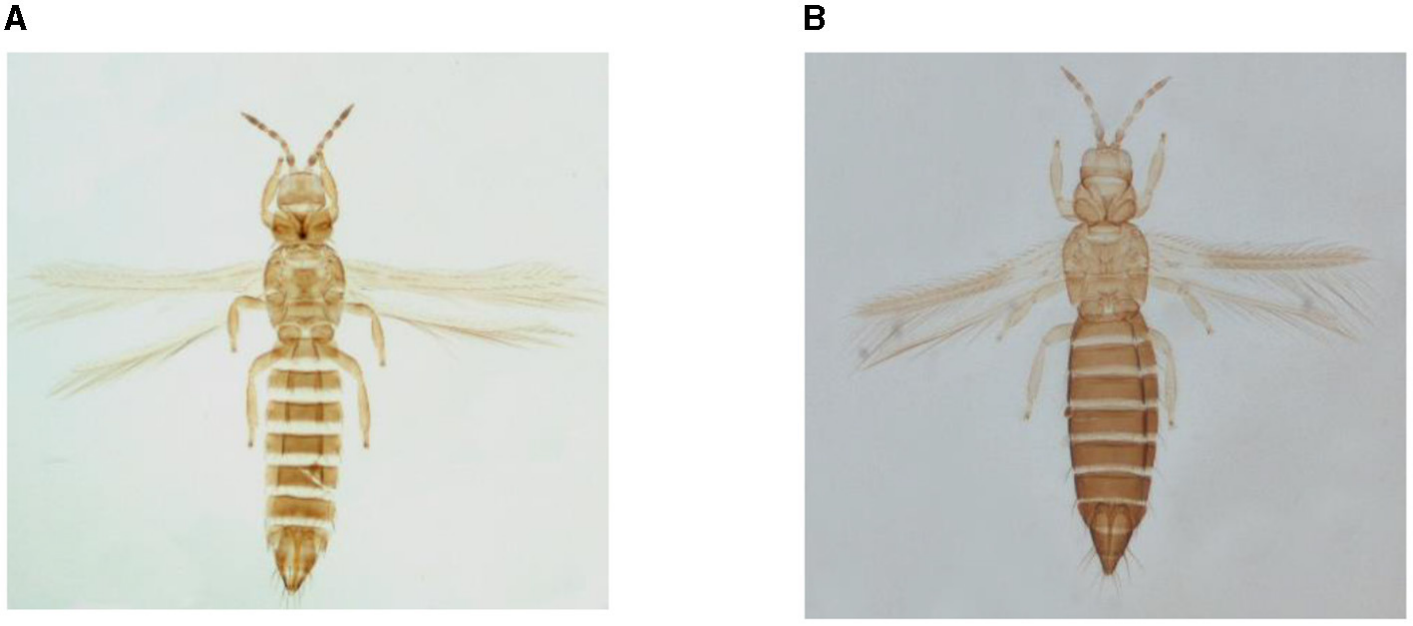
*Thrips* sp. collected from onion fields. **(A)** *Thrips tabaci*. **(B)** *Thrips parvispinus*.

Thrips infesting onion crop (*Thrips tabaci* and *Thrips parvispinus*) at fields of CCS Haryana Agricultural University, Hisar, Haryana (290 10'N latitude and 750 46'E longitude) during *Rabi* 2021–22 were collected by dislodging them from infested leaves over a white paper sheet. The thrips specimens were identified up to species level following standard taxonomic keys (Mound, 2005). Zoological Survey of India, Kolkata (West Bengal) and ICAR – National Bureau of Agricultural Insect Resources, Bengaluru (Karnataka) NBAIR, Bengaluru (Karnataka) also confirmed identification with conclusive identity report as *Thrips tabaci* (Figure 1A) and *Thrips parvispinus* (Figure 1B). Further the molecular characterization of thrips was accomplished through amplication of mt COX 1 gene (Buckman et al., 2013) and the resulting sequences were submitted to National Center for Biotechnology Information (NCBI) for obtaining accession number.

### Maintenance and culturing of thrips

To establish the colony, *Thrips tabaci* and *Thrips parvispinus* were cultured in glass jars with lids containing fine mesh gauze for aeration at ambient temperature. The colonies of *Thrips tabaci* were maintained on onion (Hisar Onion 2) while that of *Thrips parvispinus* on chili. The thrips were allowed to feed *ad libitum*. Four to five fresh leaf pieces were fed every week to provide fresh foliage for continuous rearing. All the experiments were performed using synchronized larval population, starved 24 h prior to experiment.

### Induction of injury and inoculation of *Alternaria porri* isolates

**Figure 2:**
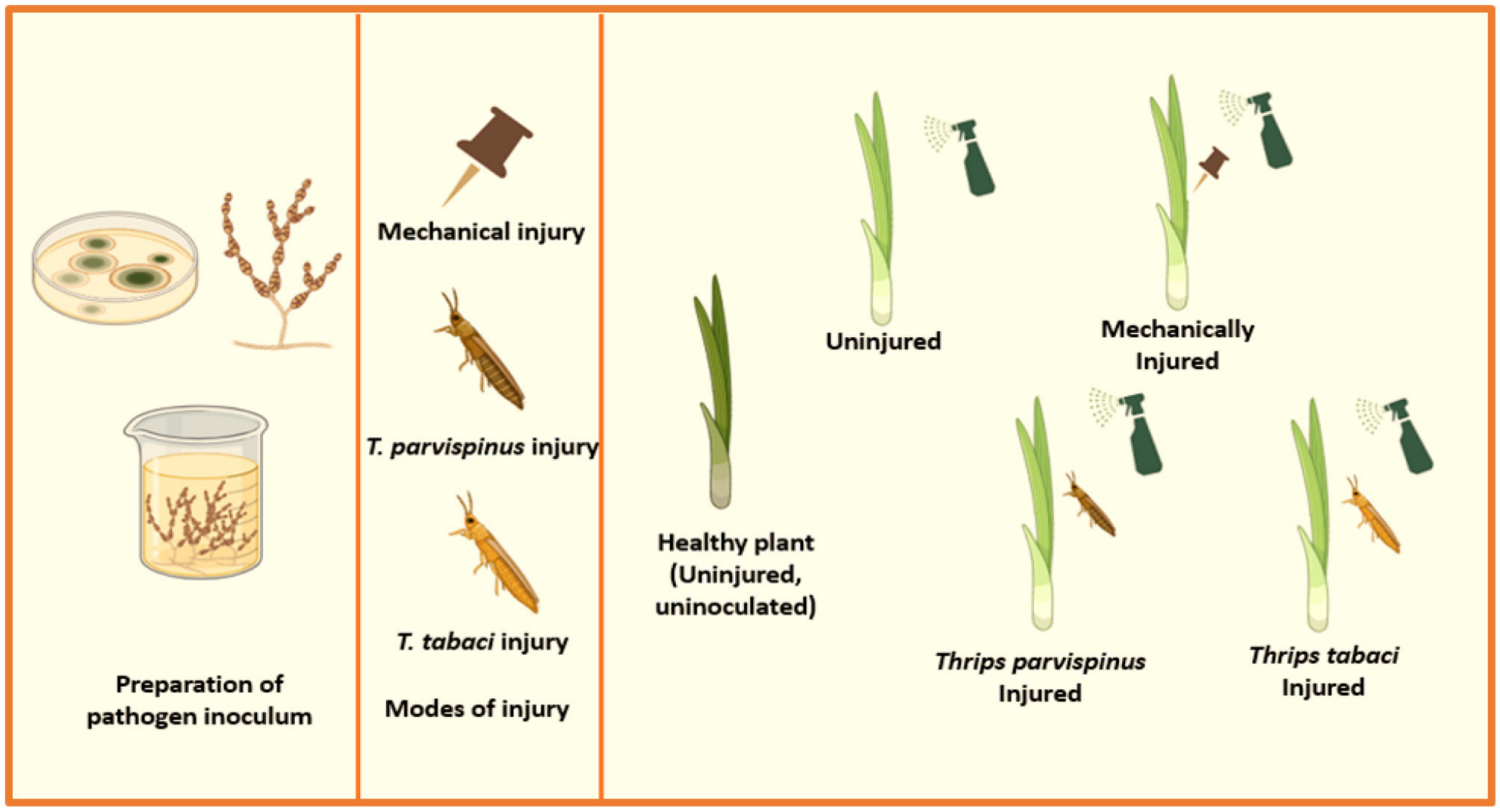
Pictorial presentation of experimental setup.

The pot experiments were conducted under screen house conditions following completely randomized design (CRD). Each treatment consists of ten plants and ten replicates per treatment were maintained. Onion (Hisar Onion 2) plants were subjected to injury via three different modes: (a) *Thrips tabaci* injury through their release on onion plants (b) *Thrips parvispinus* injury through their release on onion plants (c) artificial injury through pinprick method. The plants were sprayed with conidial suspension (1 × 10^6^/ml) of four *A. porri* isolates and each plant was covered with a plastic bag for 48 h to maintain leaf wetness for germination and infection. A suitable inoculated control (pathogen inoculated on uninjured plant) and absolute control (uninjured and un-inoculated) was maintained (Figure 2).

### Purple blotch disease assessment

The plants were assigned with identification number that was used for data collection to account for inherent observer bias while taking visual observations (James, 1974). The plants were visually assessed for purple blotch disease symptoms 14 days after inoculation. The disease incidence was recorded following the formula of Tarr (1981).


$$\begin{array}{l} Disease\;incidence\;(per\;cent)\;=\frac{Number\;of\;infected\;plants}{Total\;number\;of\;plants\;observed}\\ \;\times \;100 \end{array}$$

The grading of leaves for infestation by *A. porri* was ascertained using 0–5 scale given by Sharma (1986) where 0: No disease symptoms; 1: A few spots toward tip covering 10% leaf area; 2: Several dark purplish brown patch covering up to 20% leaf area; 3: Several patches with paler outer zone covering up to 40% leaf area; 4: Leaf streaks covering up to 75% leaf area or breaking of the leaves from center; 5: Complete drying of the leaves or breaking of the leaves from center. The disease severity was calculated using formula proposed by Wheeler (1969)


$$\begin{array}{l} Disease\;severity=\frac{Sum\;of\;all\;individual\;disease\;ratings}{Number\;of\;leaves\;observed}\\ \;\times \frac{100}{Maximum\;disease\;grade} \end{array}$$

Each leaf was evaluated for total number of purple blotch lesions, average size of lesion (cm) and average length of lesion (cm^2^).

### Assessment of morphological parameters

The plants under different treatments were rated for their leaf length. The total leaf area was calculated as per Gamiely et al. (1991) using leaf length as an index of leaf area (A = −105.5 + 4.71 X leaf length in cm; R^2^ = 0.92). The leaf diameter was measured in centimeter from middle of leaf width with help of Vernier caliper.

### Assessment of physiological parameters

The photosynthetic rate (μmol CO_2_/m^2^/s) of onion plants subjected to varied treatments was recorded in bright sunshine hours using open system LCA-4 ADC portable Infrared Gas Analyser, IRGA (Analytical Developmental Company, Hoddeson, England). Hiscox and Israelstam (1979) methodology was used to determine total chlorophyll content in onion leaves. Fresh leaf samples (100 mg) were suspended in 10 ml dimethyl sulphoxide (DMSO) and kept in dark for 12 h. Absorbance was measured at 645 and 663 nm against reagent blank. Total chlorophyll content was calculated as per the equation proposed by Arnon (1999). The methodology of Dionisio-Sese and Tobita (1998) was used to calculate electrolyte leakage in onion plants subjected to stress. Briefly, freshly cut leaf disks (1 cm diameter) were rinsed 3 times with demineralized water, thereafter floated on 10 ml demineralized water for 5 h and surrounding water's electrical conductivity (ECa) was measured. The water was then heated in water bath for 50 min and after cooling the electrical conductivity (ECb) of surrounding water was again measured. Relative Stress Injury (RSI) was calculated using the following formula:


$$\begin{array}{l} RSI\;\left(per\;cent\right)=\frac{ECa}{ECb}\times 100 \end{array}$$

### Biochemical analysis of infected leaves

The plants subjected to varied stress conditions were analyzed for their biochemical constituents. To analyze total soluble phenolics (TSP) and sugars (TSS), 0.2 grams of leaf tissue (dry weight basis) was subjected to extraction using 5 ml of 80% hot methanol (v/v). The resulting mixture was then centrifuged at 10,000 rpm for 10 min, and the supernatant was subsequently utilized for further analysis. Total soluble phenolics were estimated using the Folin-Ciocalteu (FC) reagent (Bray and Thorpe, 1954) and total soluble sugars were estimated using the phenol-sulfuric acid method (DuBois et al., 1956) with slight modifications as described by Saini et al. (2023). For estimation of total proteins, leaf tissue (0.2 g) was homogenized in 3 ml of 0.1 M potassium phosphate buffer (pH 7.0) and centrifuged for 15 min at 10,000 rpm (4 °C). The total soluble protein in the supernatant was precipitated by adding 20% trichloroacetic acid (TCA) overnight. The content was centrifuged; residues were washed twice with cold acetone and re-dissolved in 0.5 ml of 0.1 N NaOH solution. The total soluble proteins were worked out following the methodology of Lowry et al. (1951) using bovine serum albumin (BSA) as the standard. For quantification of hydrogen peroxide, leaf tissue (0.2 g) was homogenized in 4 ml of pre-chilled 5% trichloroacetic acid followed by addition of 100 mg of activated charcoal and centrifuged at 7,000 rpm for 15 min. The supernatant was used to estimate hydrogen peroxide content using the protocol of Sinha (1972).

### Statistical analysis

The observations recorded were subjected to Duncan Multiple Range Test (*P* < 0.005) to separate the means using OPSTAT software (http://hau.ernet.in/OPSTAT). The correlation of disease severity index with morpho- physiological and biochemical parameters and resulting scatter plot was worked out using XLSTAT statistical software for excel (https://www.xlstat.com). To determine whether, there exist significant difference between different groups (uninjured, mechanically injured, *Thrips parvispinus* injured and *Thrips tabaci* injured), *post-hoc* Games Howell test was employed owing to unequal variances among the groups using R studio.

## Results

### Effect of injury on *Alternaria porri* parasitism

**Figure 3:**
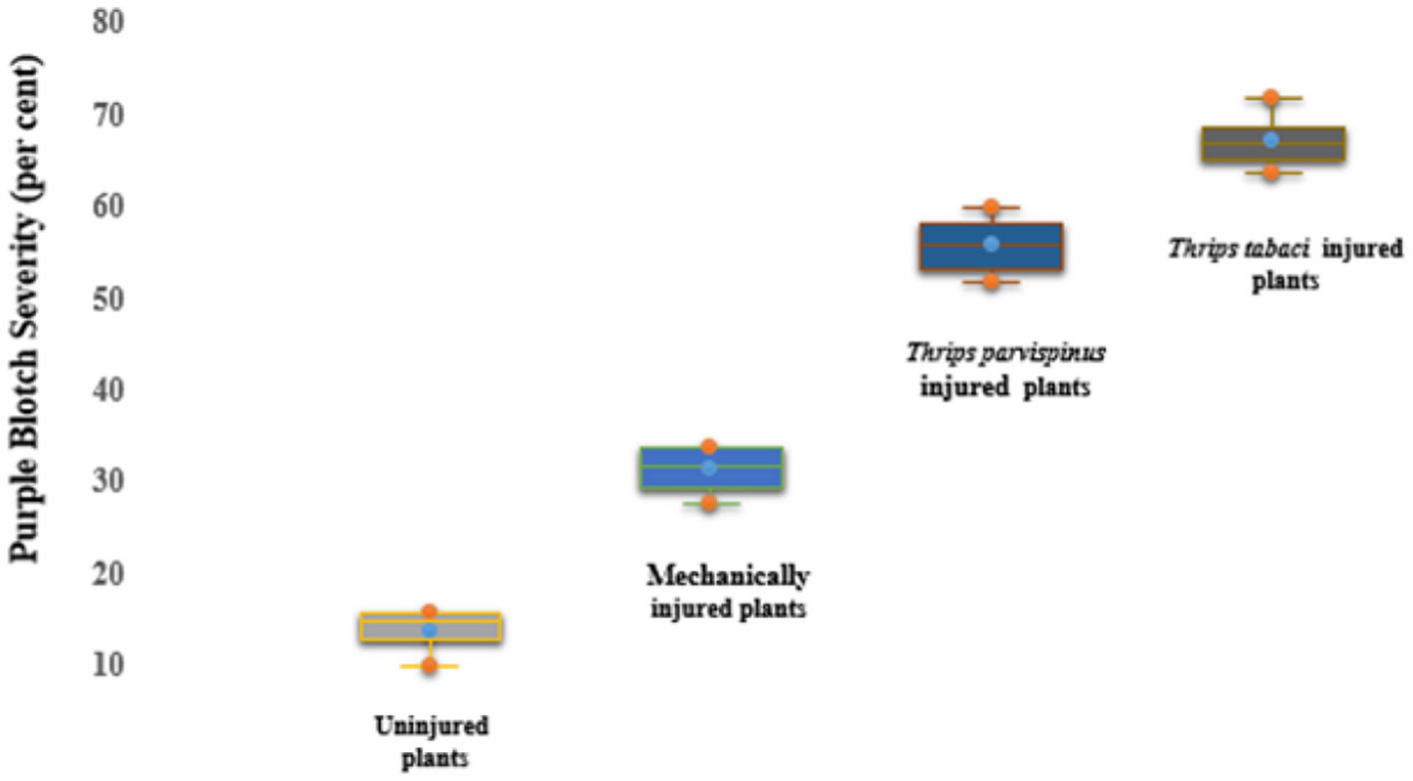
Purple blotch severity upon concomitant occurrence of injury and *Alternaria porri*.

Injury inflicted on onion plants significantly influenced the parasitism by *A. porri*. The maximum disease incidence was observed on *A. porri* inoculated on thrips injured plants exhibiting 100% disease incidence. The mechanically injured plants inoculated with *A. porri* exhibited comparatively less disease incidence spanning from 60 to 70 per cent. The minimum incidence was observed on uninjured inoculated plants presenting incidence of 30%−40 per cent. Purple blotch severity escalated in onion plants injured with thrips (Figure 3). The maximum disease severity was recorded in plants exposed to *Thrips tabaci* and inoculated with *A. porri* (64%−72%) followed by plants rendered to feeding by *Thrips parvispinus* (52%−60%). The mechanically injured inoculated plants exhibited moderate purple blotch severity (28%−52%). The minimum severity was recorded in uninjured inoculated plants (10%−16%). No considerable variation was observed with regard to different isolate of *A. porri*; however Ap 4 and Ap 3 isolate exhibited more pronounced levels of severity.

### Effect of injury and *Alternaria porri* parasitism on symptom expression

The symptom expression in form of light to dark colored lesions giving targeted board appearance were prominent on leaves in all the inoculated treatments, their number, length and size however exhibited considerable variations (Table 1). The comparison among treatments that received no injury or mechanical injury with those received thrips injury revealed that plants subjected to thrips injury had more number of lesions per plant (5.20–7.60) compared to the former ones (2.20–4.40). The average lesion length and average lesion size increase followed the similar trend. Uninjured plants had minimum lesion length (0.62–0.70 cm) and area (0.23–0.40 cm^2^), followed by mechanically injured plants where considerable increase in average lesion length (2.12–2.74 cm) and average lesion size (1.30–1.99 cm^2^) was observed. Among thrips infested plants, *Thrips tabaci* injured plants showed maximum average lesion length (3.92–4.04 cm) and size (2.88–3.31 cm^2^) followed by *Thrips parvispinus* showing average lesion length (3.00–3.36 cm) and size (2.34–2.89 cm^2^). The impact of *A. porri* isolate inoculation also had a significant impact on symptomatology expression. Games Howell test also indicated that injured plants exhibited significantly higher number of lesions per plant (Supplementary Figure A1), average lesion length (Supplementary Figure A2) and average lesion size (Supplementary Figure A3) (*p* < 0.001) compared to uninjured and control.

**Table 1:** Effect of thrips injury on purple blotch symptom expression.

**Injury**	***A. porri* isolate**	**No. of lesions/plants**	**Average lesion length (cm)**	**Average lesion size (cm^2^)**
Uninjured	Ap 1	2.20^j^ ± 0.20	0.62^g^ ± 0.07	0.23^h^ ± 0.04
	Ap 2	2.40^ij^ ± 0.24	0.62^g^ ± 0.03	0.26^h^ ± 0.09
	Ap 3	2.60^ij^ ± 0.24	0.68^g^ ± 0.08	0.28^h^ ± 0.07
	Ap 4	3.00^ij^ ± 0.31	0.70^g^ ± 0.07	0.40^h^ ± 0.05
Mechanical injury	Ap 1	3.20^hi^ ± 0.37	2.12^f^ ± 0.08	1.30^g^ ± 0.14
	Ap 2	4.00^gh^ ± 0.31	2.30^ef^± 0.03	1.42^fg^ ± 0.21
	Ap 3	4.20^g^ ± 0.37	2.44^e^ ± 0.05	1.79^ef^ ± 0.12
	Ap 4	4.40^fg^ ± 0.40	2.74^d^ ± 0.06	1.99^de^ ± 0.26
*T. parvispinus* injury	Ap 1	5.20^ef^ ± 0.37	3.00^c^ ± 0.10	2.34^cd^ ± 0.19
	Ap 2	5.60^de^ ± 0.24	3.16^bc^ ± 0.17	2.43^bcd^ ± 0.19
	Ap 3	5.80^de^ ± 0.37	3.18^bc^ ± 0.13	2.79^abc^ ± 0.11
	Ap 4	6.40^bcd^ ± 0.40	3.36^b^± 0.11	2.89^ab^ ± 0.11
*T. tabaci* injury	Ap 1	6.20^cd^ ± 0.37	3.92^a^ ± 0.08	2.88^ab^ ± 0.30
	Ap 2	6.80^abc^ ± 0.37	3.94^a^ ± 0.09	3.02^a^ ± 0.25
	Ap 3	7.20^ab^ ± 0.37	3.98^a^ ± 0.12	3.21^a^ ± 0.30
	Ap 4	7.60^a^ ± 0.51	4.04^a^ ± 0.12	3.31^a^ ± 0.17
Control		0.00^k^ ± 0.00	0.00^k^ ± 0.00	0.00^h^ ± 0.00

Means within a column followed by the same letter(s) are not significantly different (*P* < 0.05, Duncan's Multiple Range Test).

### Effect of injury and *Alternaria porri* parasitism on morphological parameters

The impact of concomitant occurrence of injury and *A. porri* parasitism on various morphological characters is presented in Table 2. Plants receiving both thrips injury and *A. porri* inoculation had shorter leaf lengths (24.00–26.60 cm) and lesser total leaf area (7.54–19.78 cm^2^) than plants that received mechanical injury or no injury where leaf length varied between 27.00 and 28.80 cm and leaf area varied from 21.67 to 30.14 cm^2^. Games Howell test also indicated that injured plants exhibited significantly shorter leaf length leaf and leaf area (*p* < 0.001) compared to uninjured and control plants (Supplementary Figures A4, A5). The observations on leaf diameter under different injuries and pathogen inoculation didn't exhibit significant differences (Table 2), also revealed by Games Howell test (Supplementary Figure A6).

**Table 2:** Effect of injury and *A. porri* parasitism on morphological characters.

**Injury**	***A. porri* isolate**	**Leaf length (cm)**	**Leaf area**	**Leaf length (cm)**
Uninjured	Ap 1	28.80^a^ ± 0.20	30.14^a^ ± 0.94	1.15^a^ ± 0.02
	Ap 2	28.60^ab^ ± 0.60	29.20^ab^ ± 2.82	1.15^a^ ± 0.02
	Ap 3	28.40^abc^ ± 0.51	28.26^abc^ ± 2.40	1.15^a^ ± 0.02
	Ap 4	28.20^abc^± 0.20	27.32^abc^ ± 0.94	1.15^a^ ± 0.03
Mechanical injury	Ap 1	28.00^abcd^ ± 0.44	26.38^abcd^ ± 2.10	1.15^a^ ± 0.03
	Ap 2	27.80^abcde^ ± 0.49	25.43^abcde^ ± 2.30	1.15^a^ ± 0.03
	Ap 3	27.20^bcdef^ ± 0.66	22.61^bcdef^ ± 3.12	1.15^a^ ± 0.03
	Ap 4	27.00^cdef^ ± 0.31	21.67^cdef^ ± 3.33	1.15^a^ ± 0.03
*T. parvispinus* injury	Ap 1	26.60^defg^ ± 0.51	19.78^defg^ ± 2.40	1.14^a^ ± 0.03
	Ap 2	26.40^efgh^ ± 0.67	18.84^efgh^ ± 3.19	1.14^a^ ± 0.03
	Ap 3	25.80^fghi^ ± 0.37	16.01^fghi^ ± 1.76	1.14^a^ ± 0.03
	Ap 4	25.20^ghij^ ± 0.37	13.19^ghij^ ± 1.76	1.14^a^ ± 0.03
*T. tabaci* injury	Ap 1	25.00^hij^ ± 0.70	12.25^hij^ ± 3.33	1.14^a^ ± 0.03
	Ap 2	24.80^ij^ ± 0.37	11.30^ij^ ± 1.76	1.14^a^ ± 0.03
	Ap 3	24.20^j^ ± 0.20	8.48^j^ ± 0.94	1.14^a^ ± 0.03
	Ap 4	24.00^j^ ± 0.44	7.54^j^ ± 2.10	1.14^a^ ± 0.03
Control		29.20^a^ ± 0.49	32.03^a^ ± 2.30	1.15^a^ ± 0.02

Means within a column followed by the same letter(s) are not significantly different (*P* < 0.05, Duncan's Multiple Range Test).

**Figure 4:**
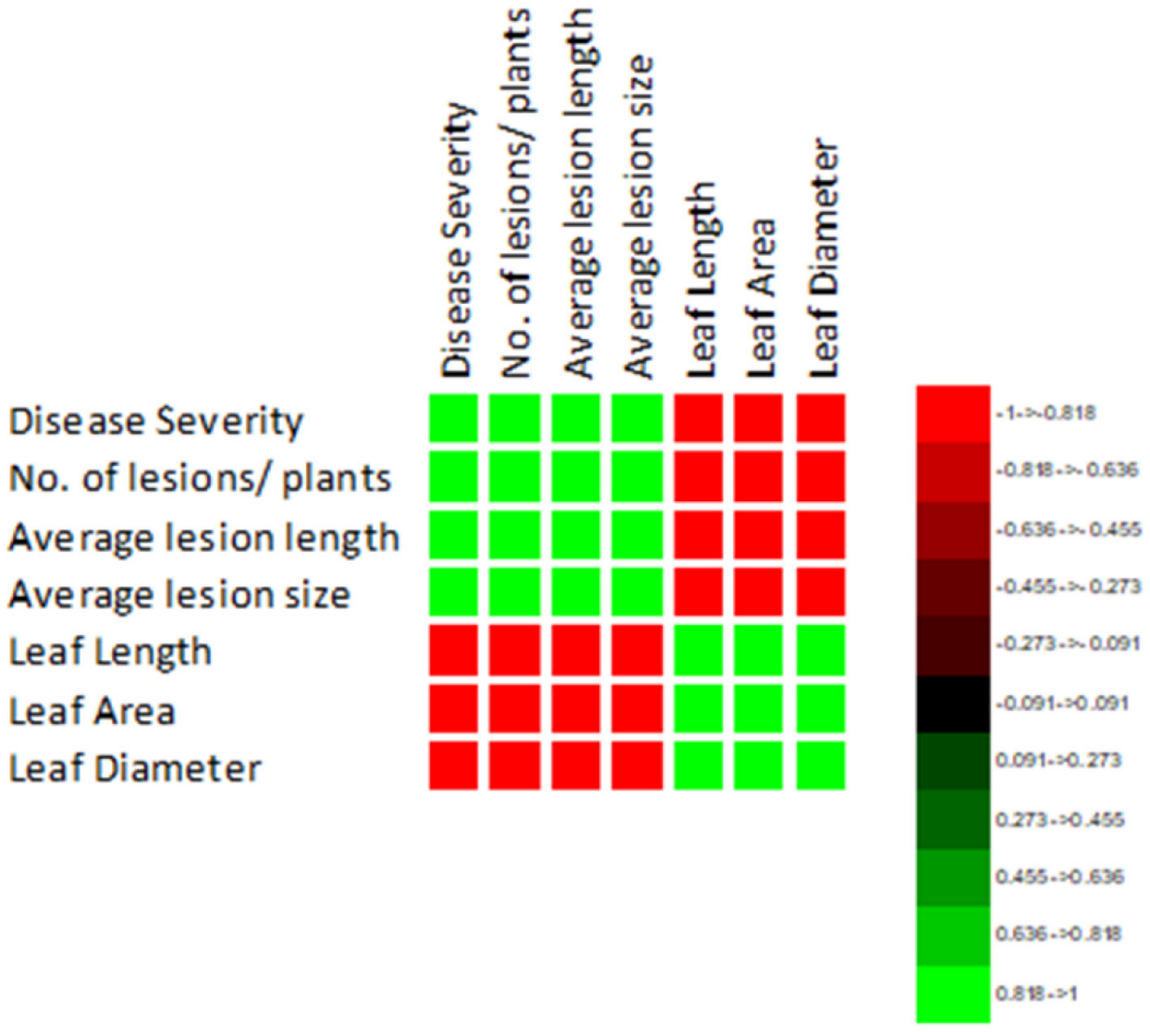
Correlation matrix indicating relation of purple blotch disease severity with disease assessment and morphological parameters.

**Figure 5:**
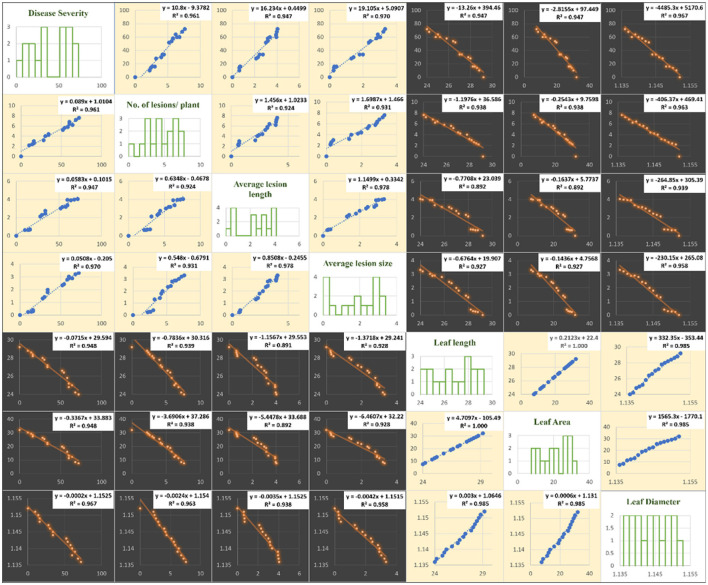
Matrix of plots of disease assessment and morphological parameters: histogram for various parameters under study (diagonal) and a scatter plot for all combinations. Blue color in scatter plots indicate positive correlation while orange color indicates negative correlation.

The correlation of purple blotch disease severity with disease assessment and morphological parameters presented in Figure 4 indicated a significant positive correlation with number of lesions per plant (0.980), average lesion length (0.973) and average lesion size (0.985) while negative correlation with leaf length (−0.974), leaf area (−0.974) and leaf diameter (−0.983). The high coefficient of determination (Figure 5) was revealed between onion purple blotch severity and various parameters viz., number of lesions per plant (0.961), average lesion length (0.947), average lesion size (0.970), leaf length (0.948), leaf area (0.948) and leaf diameter (0.967). The points in scatter plot are tightly clustered around the trend line indicating the strength of relation between the variables (Figure 5).

### Effect of injury and *Alternaria porri* parasitism on physiological parameters

#### Photosynthetic rate

**Figure 6:**
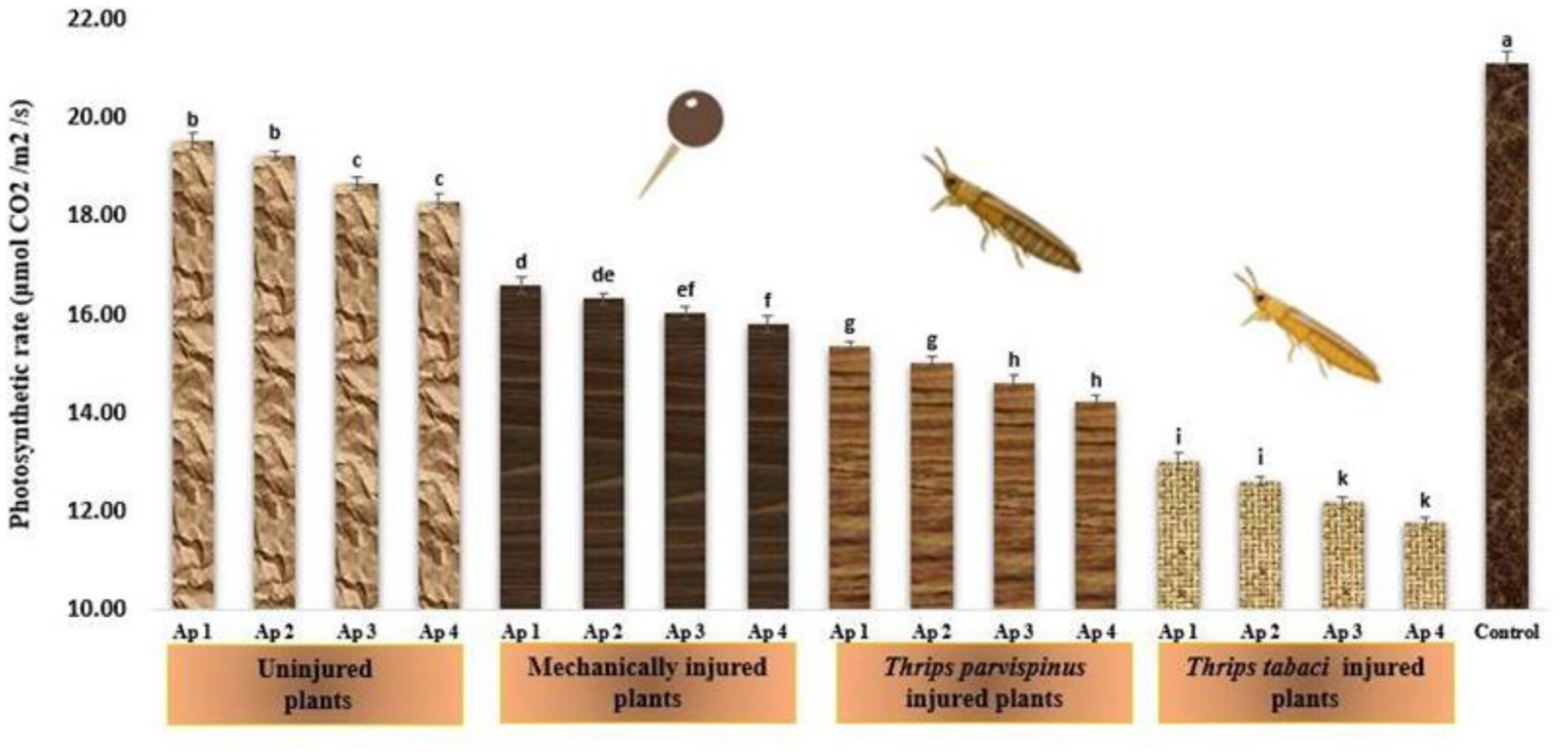
Impact of injury and *Alternaria porri* parasitism on photosynthetic rate. Bars indicate the mean values; error bars represent the standard error. Bars with different letter(s) are significantly different from each other (*p* = 0.005) according to Duncan Multiple Range Test.

The photosynthetic rate of onion plants underscores significant variations with type of injury inflicted and pathogen isolate inoculated on the onion plant (Figure 6). The highest photosynthetic rate (21.13 μmolCO_2_/m^2^/s) was observed in healthy (control) plants while it started decreasing as the level of stress was enhanced. Amongst the various injury treatments, *Thrips tabaci* injured plants exhibited the lowest photosynthetic rate (ranging from 11.77 to 13.03 μmol CO_2_/m^2^/s), followed by *Thrips parvispinus* injured plants (14.21 to 15.36 μmol CO_2_/m^2^/s) and mechanically injured plants (15.81 to 16.61 μmol CO_2_/m^2^/s) while the plants with no injury exhibited photosynthetic rate varying from 18.32 to 19.53 μmol CO_2_/m^2^/s. Within each injury level, the inoculation with different isolates of *Alternaria porri* also exhibited significant variation, with Ap 4 exhibiting highest decrease in photosynthetic rate, followed by Ap 3, Ap 2 and Ap 1. Games Howell test also indicated that injured plants exhibited significantly lower photosynthetic rate (*p* < 0.001) compared to uninjured and control (Supplementary Figure A7).

#### Chlorophyll content

**Figure 7:**
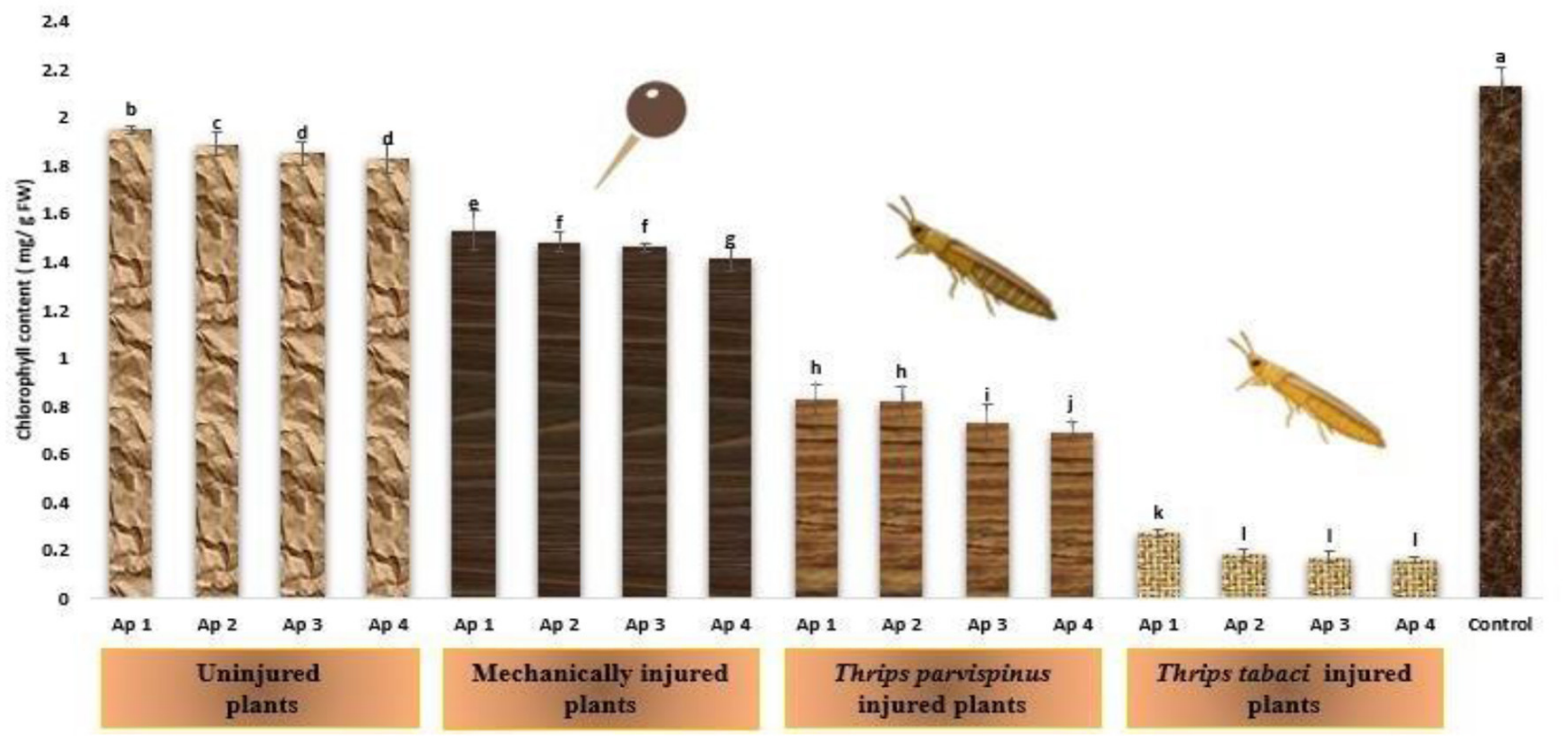
Impact of injury and *Alternaria porri* parasitism on chlorophyll content. Bars indicate the mean values; error bars represent the standard error. Bars with different letter(s) are significantly different from each other (*p* = 0.005) according to Duncan Multiple Range Test.

The drastic transformations that stress inflict on chlorophyll content of onion plants is presented in Figure 7, implicating that with escalating stress level, the content of chlorophyll exhibited remarkable decline. The healthy (control) plants exhibited highest chlorophyll content (2.13 mg/g FW). The uninjured inoculated plants exhibited chlorophyll content spanning from 1.83 to 1.95 mg/g FW while that in mechanically injured plants varied from 1.41 to 1.53 mg/g FW. *Thrips parvispinus* injured plants also exhibited reduction in chlorophyll content (0.69 to 0.83 mg/g FW) and the least chlorophyll content was observed in plants subjected to prior feeding by *Thrips tabaci* (0.16 to 0.27 mg/g FW). Within each injury level, the inoculation with different isolates of *Alternaria porri* also exhibited significant variation, with Ap 4 exhibiting highest decrease in photosynthetic rate, followed by Ap 3, Ap 2 and Ap 1. The results of Games Howell test also indicated the significance of the finding that injured plants with reduced chlorophyll content stands apart exhibiting significantly lower chlorophyll content (*p* < 0.001) compared to uninjured and control plants (Supplementary Figure A8).

#### Relative stress injury

**Figure 8:**
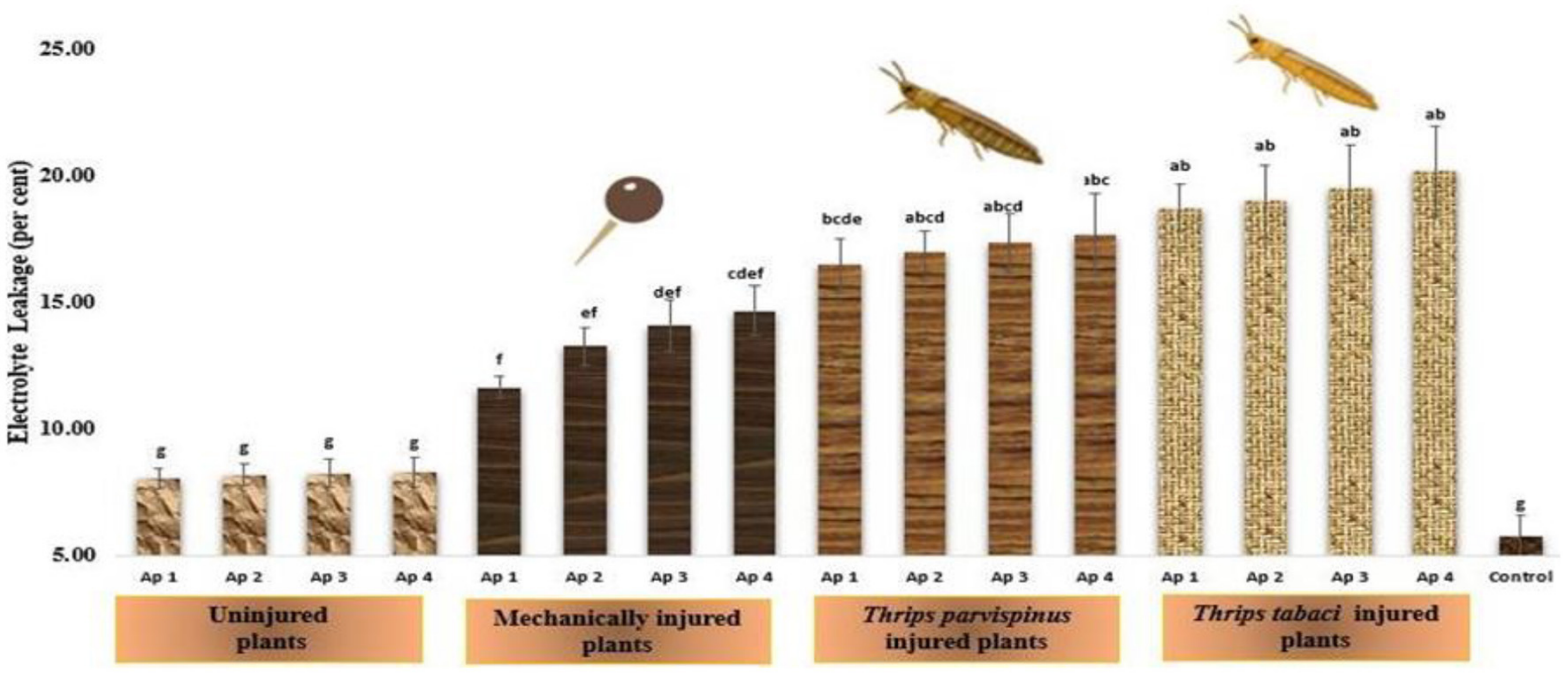
Impact of injury and *Alternaria porri* parasitism on relative stress injury. Bars indicate the mean values; error bars represent the standard error. Bars with different letter(s) are significantly different from each other (*p* = 0.005) according to Duncan Multiple Range Test.

The vulnerability on onion plants to relative stress injury (RSI) upon infliction of injury and inoculation of pathogen is reflected in Figure 8. RSI exhibit an array of variations among different treatments, however the healthy plants (5.78%) and uninjured inoculated plants (8.08%−8.30%) exhibited least relative stress injury, which was observed statistically at par in accordance with Duncan Multiple Range Test, indicating a fairly consistent response in the particular context. The injured inoculated plants exhibited significant variations for relative stress injury with maximum values recorded on plants exposed to *Thrips tabaci* spanning from 18.74 to 20.18 per cent, found statistically similar regardless of the isolate used. *Thrips parvispinus* injured plants (16.49%−17.68%) unfolded the little statistical differences with the isolate of *Alternaria porri* inoculated. The mechanically injured plants presented the significant variations in relative stress injury upon inoculation with different isolates, the maximum injury observed upon inoculation with Ap 4 (14.68%), followed by Ap 3 (14.13%), Ap 2 (13.28%) and Ap 1 (11.67%). Games Howell test also indicated that injured plants exhibited significantly higher levels of stress injury (*p* < 0.001) compared to uninjured and control (Supplementary Figure A9).

### Effect of injury and *Alternaria porri* parasitism on biochemical changes

The response of onion plant to stress manifest through dynamic alteration in biochemical alterations in total phenolics, total soluble sugars, total soluble proteins, and hydrogen peroxide plant.

#### Total phenolic content

**Figure 9:**
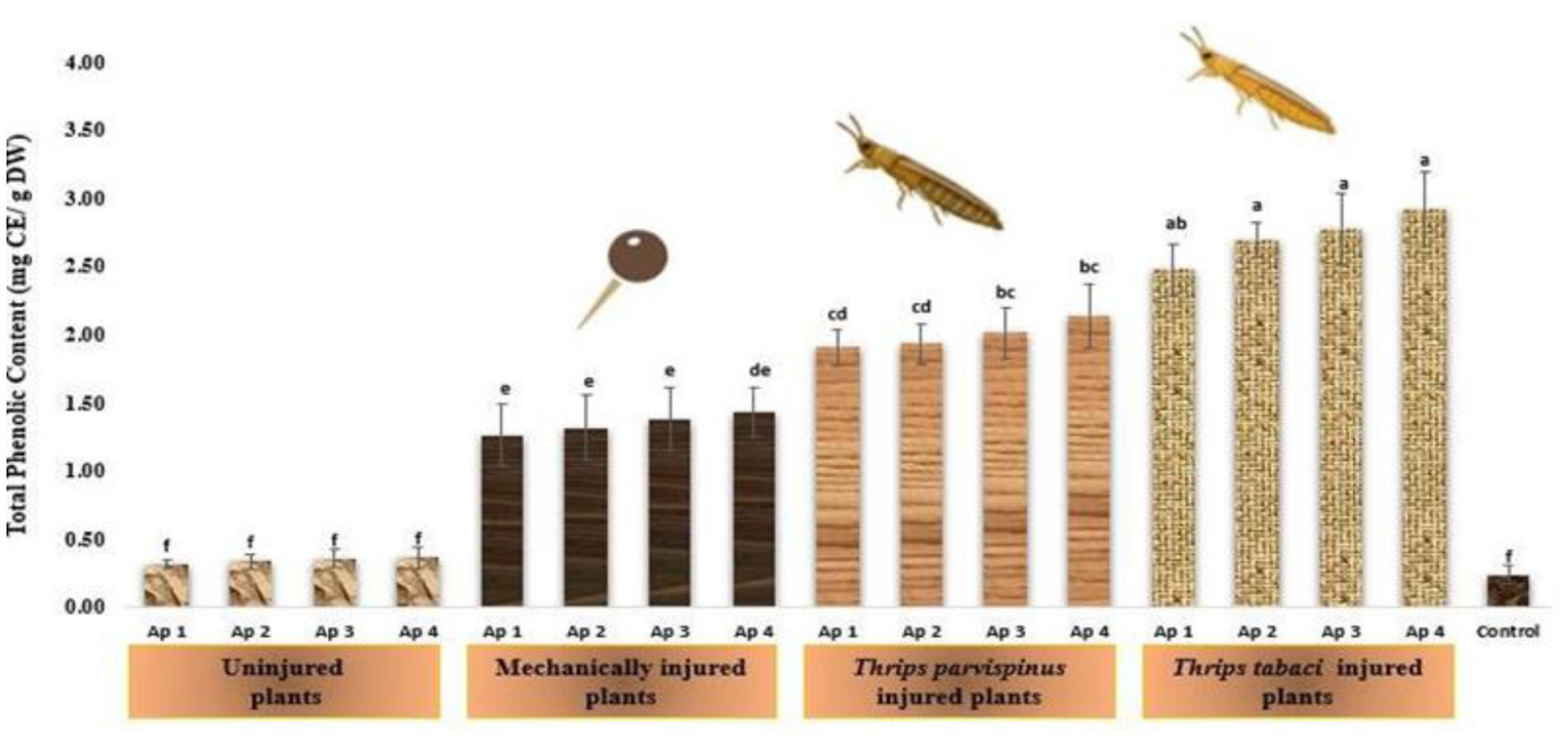
Impact of injury and *Alternaria porri* parasitism on total phenol content. Bars indicate the mean values; error bars represent the standard error. Bars with different letter(s) are significantly different from each other (*p* = 0.005) according to Duncan Multiple Range Test.

Total phenolic content of onion leaves varied significantly with type of injury inflicted on plant. It was evident that with increasing stress level, the level of total phenolic content increased from healthy plants to insect injured plants (Figure 9). The highest phenolic content was observed in *Thrips tabaci* injured plants followed by inoculation with Ap 4 (2.93 mg CE/g DW), Ap 3 (2.78 mg CE/g DW), Ap 2 (2.70 mg CE/g DW) and Ap 1 (2.48 mg CE/g DW). The similar trend was observed when plant priorly exposed to *Thrips parvispinus* were subjected to pathogen inoculation, exhibiting the values of 2.14 mg CE/g DW, 2.01 mg CE/g DW, 1.93 mg CE/g DW and 1.91 mg CE/g DW with respective isolates. The mechanically injured plants upon pathogen inoculation pathogen also exhibited elevated level of total phenolic content varying from 1.27 to 1.43 mg CE/g DW. However, it is worth noting that uninjured inoculated plants (0.32- 0.36 mg CE/g DW) and healthy plants (0.24 mg CE/g DW) exhibited statistically similar values for total phenolic content as revealed by Duncan Multiple Range Test (*p* < 0.005), an implication to consistency in response of onion plants to unstressed and pathogen stressed plants. The results of Games Howell test implicated that total phenolic content in onion leaves (Supplementary Figure A10) exhibit significant differences (*p* < 0.001) with type of injury inflicted on the plant.

#### Total soluble sugars

**Figure 10:**
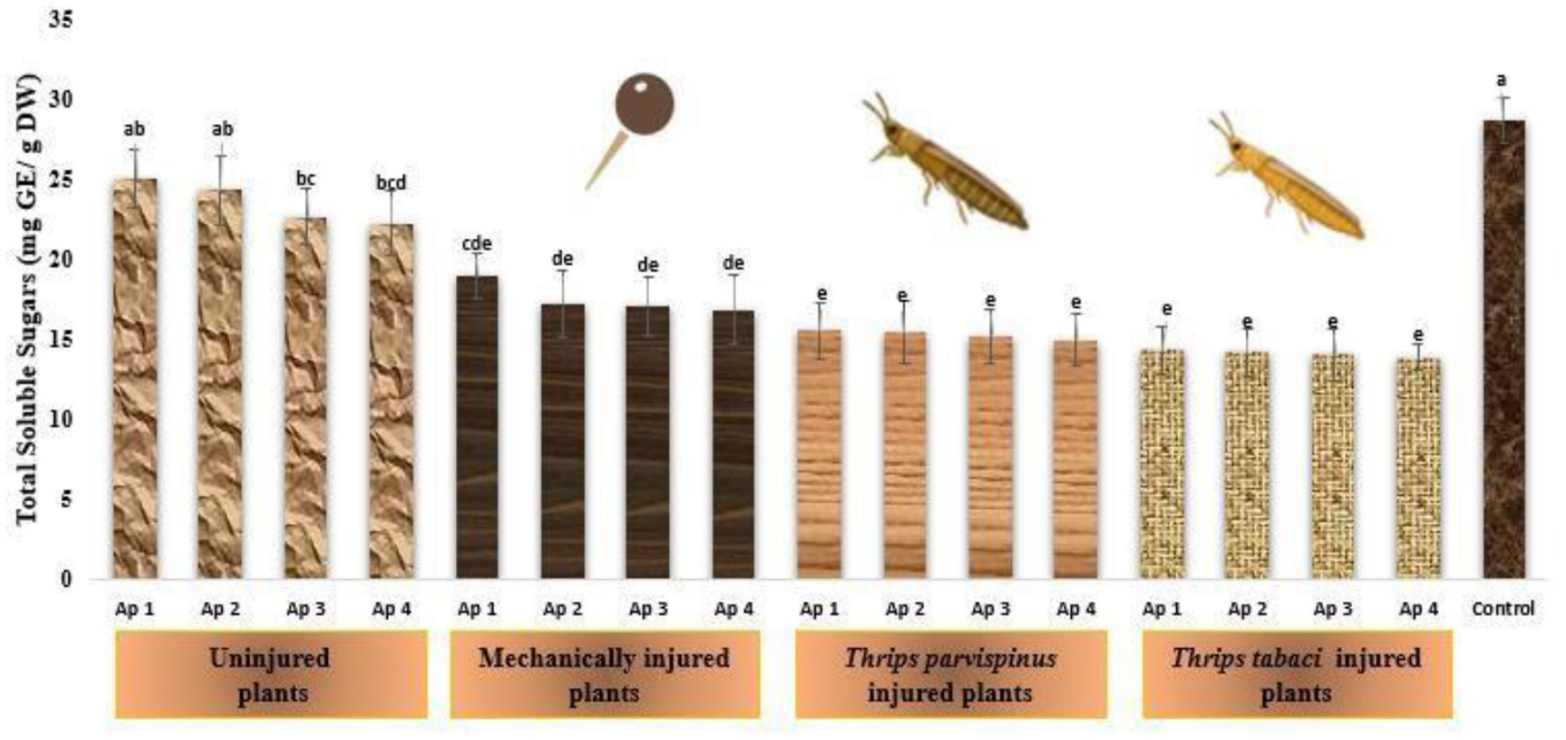
Impact of injury and *Alternaria porri* parasitism on total soluble sugars. Bars indicate the mean values; error bars represent the standard error. Bars with different letter(s) are significantly different from each other (*p* = 0.005) according to Duncan Multiple Range Test.

The dynamic changes in total soluble sugars within the leaves of onion reflects their response to stress (Figure 10). The healthy onion plants exhibited remarkable higher amount of total soluble sugars (28.74 mg GE/g DW), and subsequent reduction was observed on inoculation with Ap 1 (25.07 mg GE/g DW), followed by Ap 2 (24.34 mg GE/g DW), Ap 3 (22.68 mg GE/g DW) and Ap 4 (22.25 mg GE/g DW). Further with induction of mechanical injury and subsequent *Alternaria porri* inoculation, the total soluble sugar content decrease, varying from 16.86 to 18.97 mg GE/g DW. The total soluble sugars in onion plants exposed prior to *Thrips parvispinus* (15.00–15.62 mg GE/g DW) and *Thrips tabaci* (13.93–14.38 mg GE/g DW), subsequently inoculated with pathogen was statistically similar, indicating a fairly consistent response in the particular context. The results of Games Howell test implicated that total soluble in onion leaves (Supplementary Figure A11) exhibit significant differences (*p* < 0.001) with type of injury inflicted on the plant.

#### Total soluble proteins

**Figure 11:**
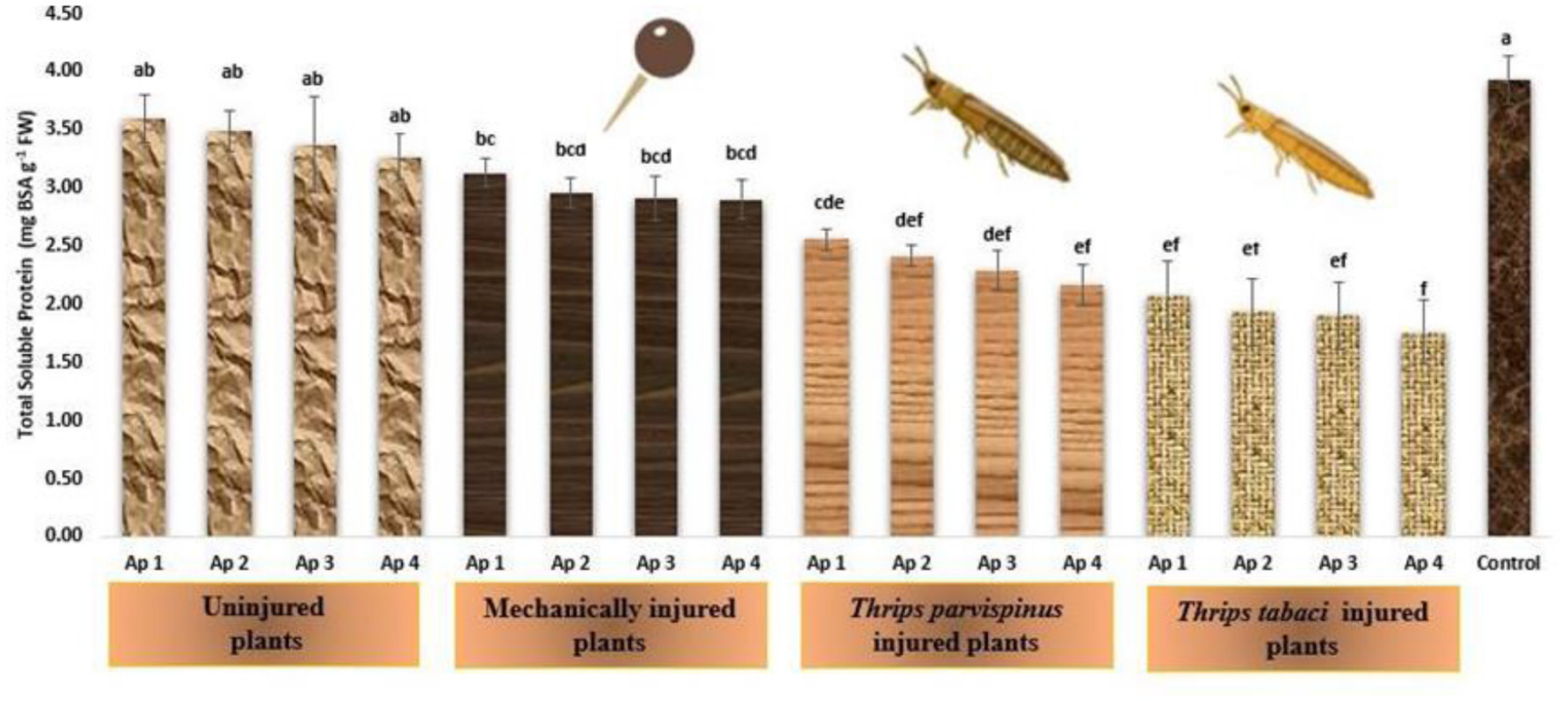
Impact of injury and *Alternaria porri* parasitism on total protein content. Bars indicate the mean values; error bars represent the standard error. Bars with different letter(s) are significantly different from each other (*p* = 0.005) according to Duncan Multiple Range Test.

The total soluble proteins in onion leaves exhibited declining trend upon encountering any kind of stress (Figure 11). The healthy onion leaves presented the highest amount of total soluble proteins (3.98 mg BSA/g FW), which exhibited declining transition upon inoculation with *Ap* 1 (3.59 mg BSA/g FW), followed by Ap 2 (3.49 mg BSA/g FW), Ap 3 (3.37 mg BSA/g FW) and Ap 4 (3.26 mg BSA/g FW), however the total soluble protein content remains statistically comparable, signifying a consistent response within this context. The mechanical injured plants following pathogen inoculation further decreased the total protein content in onion leaves (2.90–3.13 mg BSA/g FW). The prior thrips injury following *Alternaria porri* inoculation unfolds the fact that *Thrips tabaci* injured plants (1.76–2.07 mg BSA/g FW) exhibited more decline in protein content of leaves compared to *Thrips parvispinus* injured plants (2.17–2.56 mg BSA/g FW). The results of Games Howell test implicated that total soluble proteins in onion leaves (Supplementary Figure A12) exhibit significant differences (*p* < 0.001) with type of injury inflicted on the plant.

#### Hydrogen peroxide

**Figure 12:**
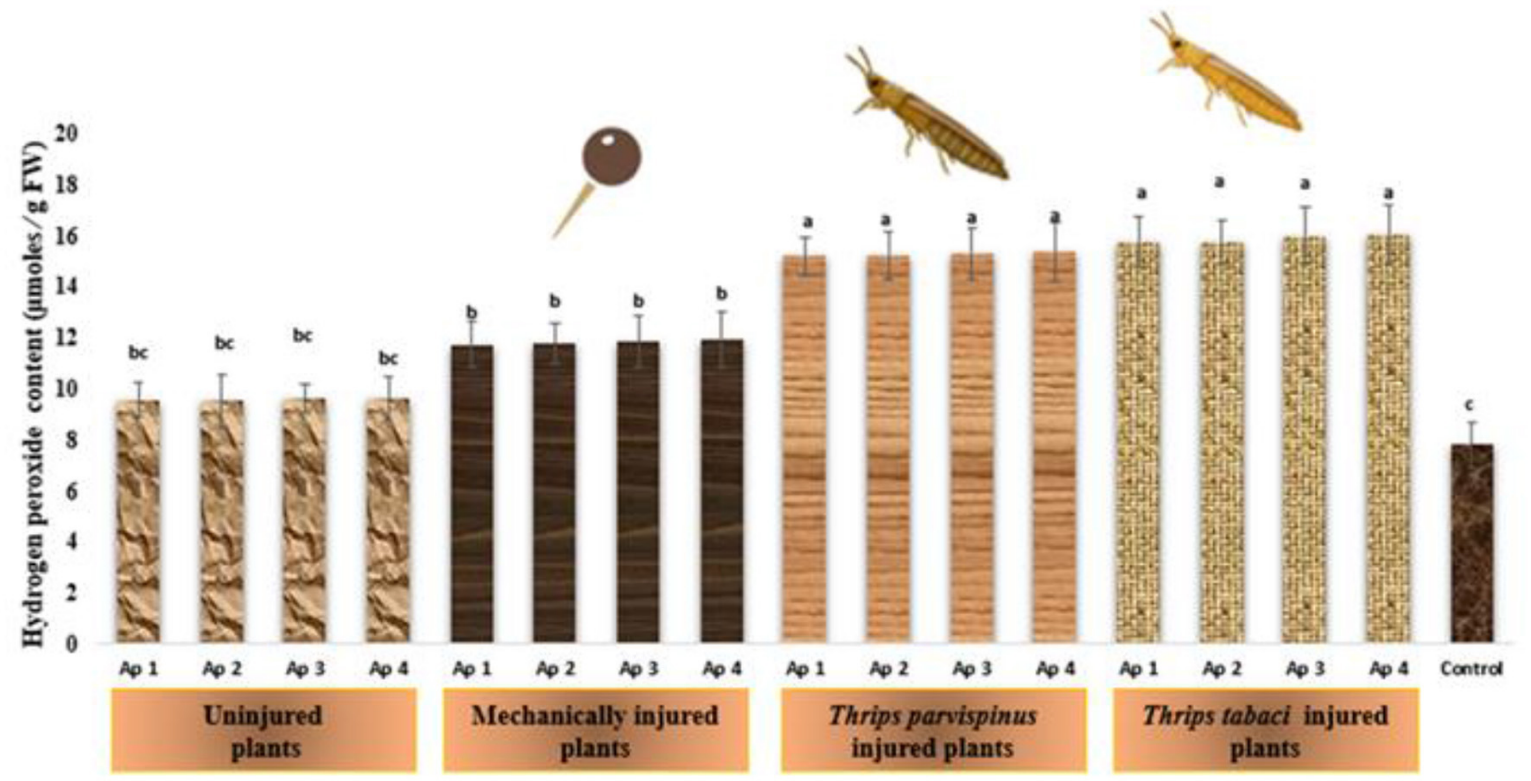
Impact of injury and *Alternaria porri* parasitism on hydrogen peroxide content. Bars indicate the mean values; error bars represent the standard error. Bars with different letter(s) are significantly different from each other (*p* = 0.005) according to Duncan Multiple Range Test.

The charismatic changes in content of hydrogen peroxide in onion plants owing to imposition of varied stress levels is depicted in Figure 12. The healthy plants signify the lowest amount of hydrogen peroxide (7.80 μ moles /g FW) in onion leaves. The combined effect of mechanical injury and *Alternaria porri* inoculation (11.73–11.93 μ moles /g FW) has more pronounced effect on hydrogen peroxide content than plants with only *Alternaria porri* inoculation (9.55–9.59 μ moles /g FW), the changes are not significantly different with different isolates inoculated as indicated by Duncan Multiple Range Test. Further intriguing findings revealed that thrips induced injury combined with *Alternaria porri* parasitism exhibit statistically similar changes in hydrogen peroxide content in onion leaves, suggesting a consistence response in this specific context. The results of Games Howell test implicated that hydrogen peroxide content in onion leaves (Supplementary Figure A13) exhibit significant differences (*p* < 0.001) with type of injury inflicted on the plant.

**Figure 13:**
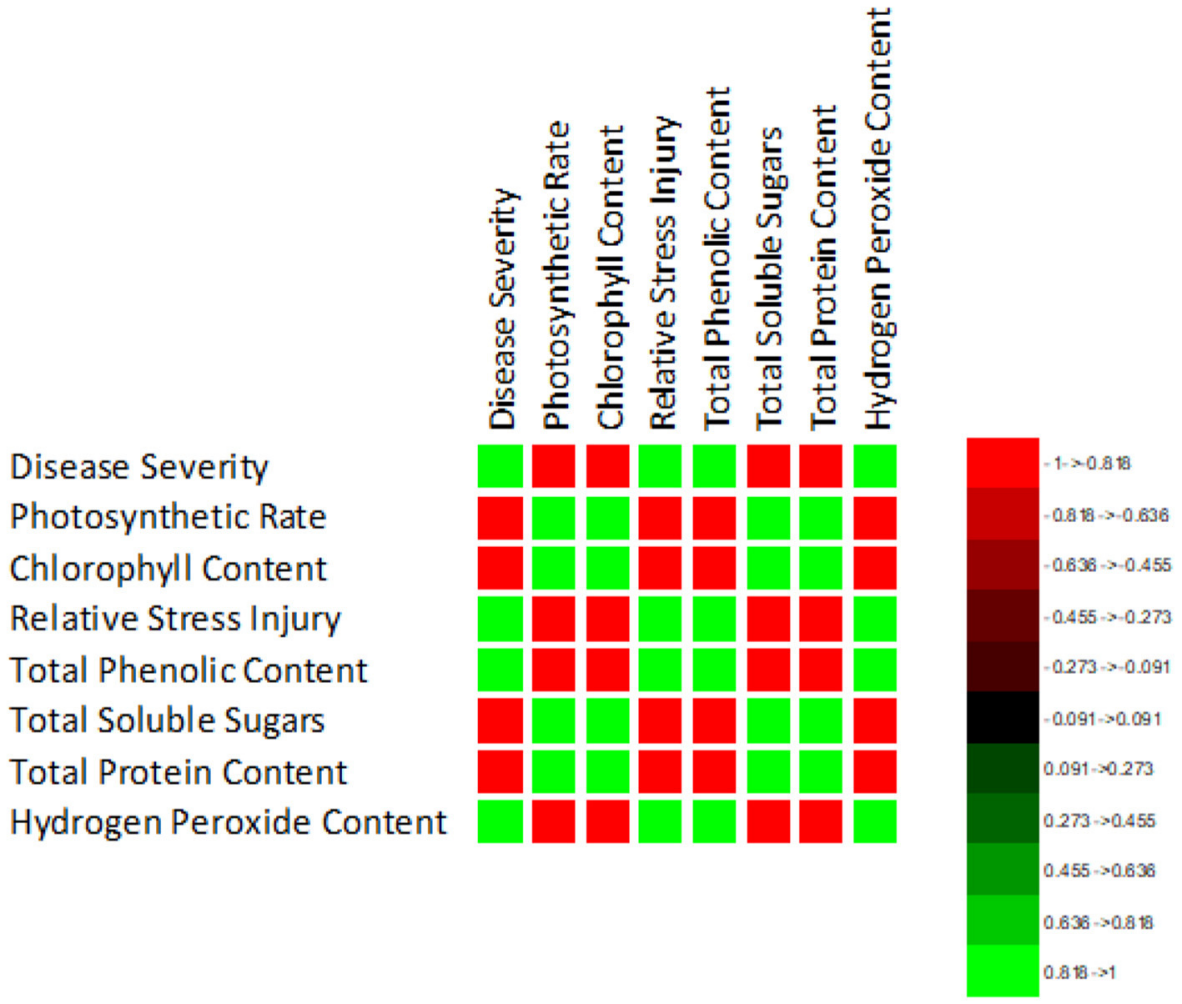
Correlation matrix indicating relation of purple blotch disease severity with physio-biochemical parameters.

**Figure 14:**
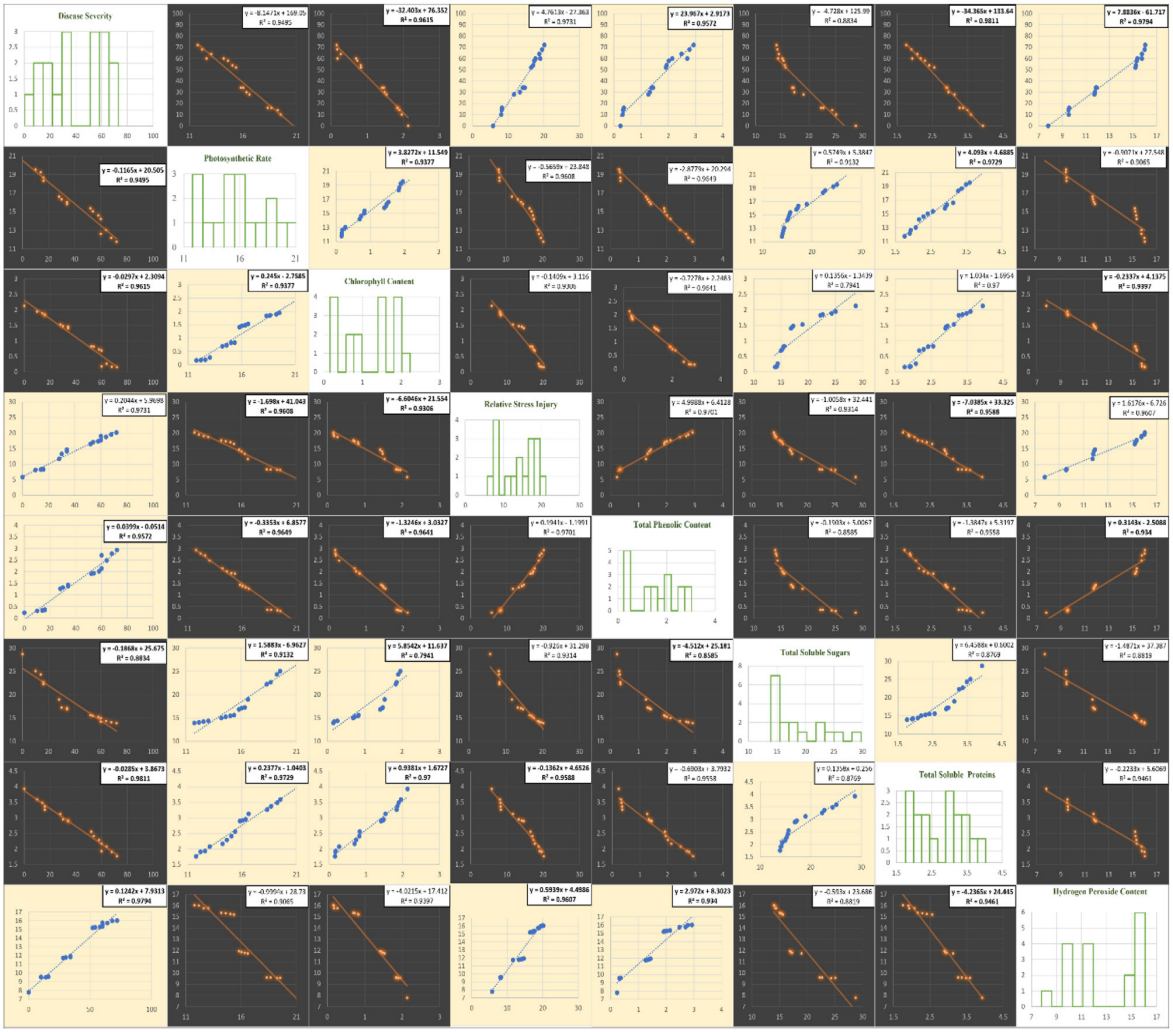
Matrix of plots of disease assessment and physio-biochemical parameters: histogram for various parameters under study (diagonal) and a scatter plot for all combinations. Blue color in scatter plots indicate positive correlation while orange color indicates negative correlation.

The correlation of purple blotch disease severity with physio biochemical parameters (Figure 13) indicated a significant positive correlation with relative stress injury (0.986), total phenolic content (0.978) and hydrogen peroxide content (0.990) while negative correlation with photosynthetic rate (−0.974), chlorophyll content (−0.981), total soluble sugars (−0.940) and total soluble proteins (−0.990). The high coefficient of determination (Figure 14) was revealed between onion purple blotch severity and various parameters viz., photosynthetic rate (0.949), chlorophyll content (0.961), relative stress injury (0.973), total phenolic content (0.957), total soluble sugars (0.883), total soluble proteins (0.981) and hydrogen peroxide content (0.979). The points in scatter plot are tightly clustered around the trend line indicating the strength of relation between the variables.

## Discussion

An injury to crop plant may be induced by chemicals or through mechanical means. Mechanical injury to above ground parts often produces obvious wounds, which may also lead to decline symptoms and at the same time serve as entry sites for disease causing pathogens. Insects are also involved in dissemination, inoculation and overwintering of plant pathogens and many wound parasites enter the plant through these openings. The present study attempted to evaluate the significance thrips feeding damage to plant in relation to onion purple development indicated that parasitism by *Alternaria porri* was enhanced in plants subject to prior thrips feeding compared to mechanically injured and uninjured plants. The purple blotch incidence was least on uninjured plants (30%−40%)while mechanically injured plants exhibited appreciable incidence reaching upto 60% and thrips injured plants exhibited 100% purple blotch incidence. The observation on development of purple blotch on uninjured plant stands contradictory to the findings of Pandotra (1964) and Bhangale and Joi (1983) who reported that no disease development occurs on uninjured plant. These cited accounts of *Alternaria porri*, not capable of entering uninjured tissue may have been dealing with *Alternaria tenius* and *Alternaria tennuisima* which were observed to penetrate only injured surface. According to Skiles (1953), three species of Alternaria (*Alternaria porri, Alternaria tenuis* and *Alternaria tennuisima*) are responsible for the disease complex known as purple and brown blotch of onion; of these three species, only *Alternaria porri* is capable of penetrating uninjured surface, which stands as supporting evidence to the observations of present study. Insect pests can influence disease incidence (Wang et al., 2008), severity (English-Loeb et al., 1999), pathogenicity, and disease symptoms (Yang et al., 2011). Thrips are known to exacerbate plant diseases caused by fungal pathogens in multiple cropping systems (Bhangale and Joi, 1983; McKenzie et al., 1993; Osekre et al., 2009). Similarly, purple blotch severity was enhanced on plants exposed to prior thrips feeding, compared to mechanically injured and uninjured plants. These findings are in corroboration with the studies conducted by McKenzie et al. (1993) who reported that *Alternaria porri* is able to penetrate uninjured tissues, although it also appears to use areas of insect damage as better alternative penetration sites, intensifying the severity of disease. The results are also in agreement to Orloff et al. (2008) who indicated that the enhancement in disease due to thrips injury may be due to creation of entry points in leaves for plant pathogens. The findings of Grode et al. (2017) also demonstrate that wounds inflicted by thrips feeding facilitate center rot development by providing entry sites to Pantoea ananatis into onion leaf tissue. Leach et al. (2020a,b) also indicated that thrips feeding on onion is associated with greater levels of *Stemphylium vesciarum* colonoization and enhanced leaf blight severity. The decreased leaf length, leaf area and enhanced lesion length and size with more purple blotch lesions due to combined effect of injury and pathogen observed in present study are strongly supported by the findings of McKenzie et al. (1993) who reported similar findings in their experiment.

Plants harbor a plethora of metabolites in order to carry complicated plant metabolic pathways in a coordinated manner during normal as well as under stressful conditions. Pathogens disrupt the normal physiological and metabolic pathways in plant (Kumar and Verma, 2018). The effect of injury and pathogen inoculation has detrimental effects on chlorophyll content and thus on photosynthetic rate. The greater decrease in chlorophyll content may be due to thrips feeding, which siphon off plant contents and consume mesophyll cells which eventually result in loss of chlorophyll and reduced photosynthetic efficiency (Boateng et al., 2014). There are reports that the insect pest infestation leads to decrease in chlorophyll content (Murugesan and Kavitha, 2010; Singh et al., 2022). Many studies demonstrated that the concentration of chlorophyll in plants decrease with infection of pathogen (Akbar et al., 2023; Smith et al., 2023; Sobhy et al., 2023). The decreased chlorophyll content in leaves following fungal invasion may be attributed to the excessive loss of water owing to increased stomatal conductance (Baghbani et al., 2019) or to the overproduction of ROS as a protective barrier which further accelerate the breakdown of chlorophyll. Electrolyte leakage is generally used as an indicator of cell membrane stability commonly used for evaluating tolerance to abiotic stress (Ilík et al., 2018), however can also be used as injury indicator for biotic stresses like pathogen infection or pest infestation. Electrolyte leakage from stressed leaves is indicative of membrane damage and subsequent cell death (Rolny et al., 2011), with electrolyte leakage increasing with damage intensity. These findings are in consistent to the observation of present findings where the electrolyte leakage was consistently increased with disease severity.

Among the various intricate pathways involved in host-pathogen interaction, the biochemical stands in forefront providing the valiant defense against the pathogen attack. Under normal conditions, optimum growth and development of plant is accomplished using the available oxygen. However, under stressful conditions, when plant attacked by any pest or pathogen, the usage of oxygen results in production of reactive oxygen species (ROS) inside the plant tissues (Singla et al., 2019), which further leads to photo-oxidative damage of biomolecules and internal cellular structures (Xie et al., 2016; Mittler, 2017). Infestation by insect pests and pathogens tend to produce H2O2 that leads to lipid peroxidation of cell membranes and the loss of vital solutes including electrolytes from the cells (Mundree et al., 2002). The enhanced activity of hydrogen peroxide following concomitant infestation of insect pest and pathogen observed in present study is in consistent with findings of many workers (Shetty et al., 2007; Singh et al., 2022), who observed marked increase in hydrogen peroxide activity following the biotic stress. The observations on decrease in protein content with enhanced damage are in consistent to the finding Amin et al. (2016) and Singh et al. (2022) who recorded decreased protein content with infestation of pests on plant. The reduction in protein content might be due to blockage of protein synthesis or degradation of protein in the host plants following the invasion. The findings on decrease in total soluble sugars after attack of pest/pathogen are in accordance with many studies (Alberto, 2014; Singh et al., 2022). The reduction in sugar content of diseased leaves may be attributed to increase in their utilization by pathogen as respiratory substrate during pathogenesis process (Ponmurugan et al., 2007). The findings, however, present a contradiction with several host-pathosystems (Herbers et al., 2000; Chou and Mansfield, 2014), where increase in total soluble sugars was recorded following pathogen infection. The variable trend in sugar levels following pathogen infection presented in different studies can be ascribed to involvement of different host pathosystems. The host-pathogen interaction unleashes a symphony of biochemical changes associated with stress signaling and thereby activating a cascade of defense responses (Debona et al., 2012; Akter et al., 2015). Phenols guard the plant against pathogen attack or ultraviolet radiation (Shahidi and Yeo, 2016). The present study indicated that type of injury inflicted on onion plant posed a significant impact on total phenolics. Stressful conditions imposed by injury and pathogen inoculation triggered defense response in plants, leading to enhanced level of phenolics. These observations on the enhancement of total phenolics with increased disease severity aligns with findings of Singh et al. (2022) and Sharma et al. (2023) who presented similar results upon attack by pathogen and pest respectively.

## Conclusion

The investigations into the interaction between onion purple blotch caused by *A. porri* and plant injury, particularly induced by thrips, has revealed a complex interplay of factors influencing disease incidence and severity. The findings underscore the importance of considering multiple components within the agro ecosystem to comprehensively understand and manage plant diseases. Plants tailor their responses to combined stress factors and exhibit many unique responses, along with some common responses. Therefore, in an attempt to recognize the impact of combined stress on plant, it is pertinent to understand the nature of such interactions. Elucidation of the mechanisms underlying these interactions among plants, pathogens and pest insects will therefore be an important step in furthering our understanding of plant defenses and the integrated management of disease and pest insects. Furthermore, the integration of entomological and pathological research emerges as a key strategy. This synergy enables more precise predictions of crop damage caused by pests, facilitating the development of sophisticated monitoring systems and targeted crop protection strategies. By bridging the gap between entomology and pathology, we can fortify our ability to address challenges at the intersection of plant health, pest management, and disease control in agricultural ecosystems.

## Data availability statement

The original contributions presented in the study are included in the article/Supplementary material, further inquiries can be directed to the corresponding author.

## Ethics statement

The animal study was approved by the Institutional Ethics Committee for Experiment on Animals, College of Agriculture, CCS Haryana Agricultural University, Hisar, Haryana, India. The study was conducted in accordance with the local legislation and institutional requirements.

## Author contributions

SS: Formal analysis, Investigation, Visualization, Writing—original draft. KR: Conceptualization, Data curation, Methodology, Project administration, Resources, Supervision, Writing—review & editing. AKS: Conceptualization, Data curation, Formal analysis, Software, Validation, Writing—original draft. RK: Methodology, Resources, Supervision, Writing—review & editing. AS: Data curation, Formal analysis, Investigation, Writing—original draft. AK: Formal analysis, Investigation, Writing—original draft. PK: Data curation, Investigation, Software, Writing—original draft. GD: Formal analysis, Investigation, Writing—original draft. MB: Data curation, Formal analysis, Validation, Writing—original draft. CM: Writing—review & editing. ML: Project administration, Resources, Supervision, Writing—review & editing. LW: Data curation, Writing—review & editing.
