# Supplementary material for: Unravelling the synergistic interaction of Thrips tabaci and newly recorded, Thrips parvispinus with Alternaria porri (Ellis.) Cif., inciting onion purple blotch
Source: Front Microbiol. 2024 Mar 5;15:1321921. doi: 10.3389/fmicb.2024.1321921 (PMC10948439; doi:10.3389/fmicb.2024.1321921)
Supplement: Supplementary file 1 [file Data_Sheet_1.docx]

**Supplementary File**


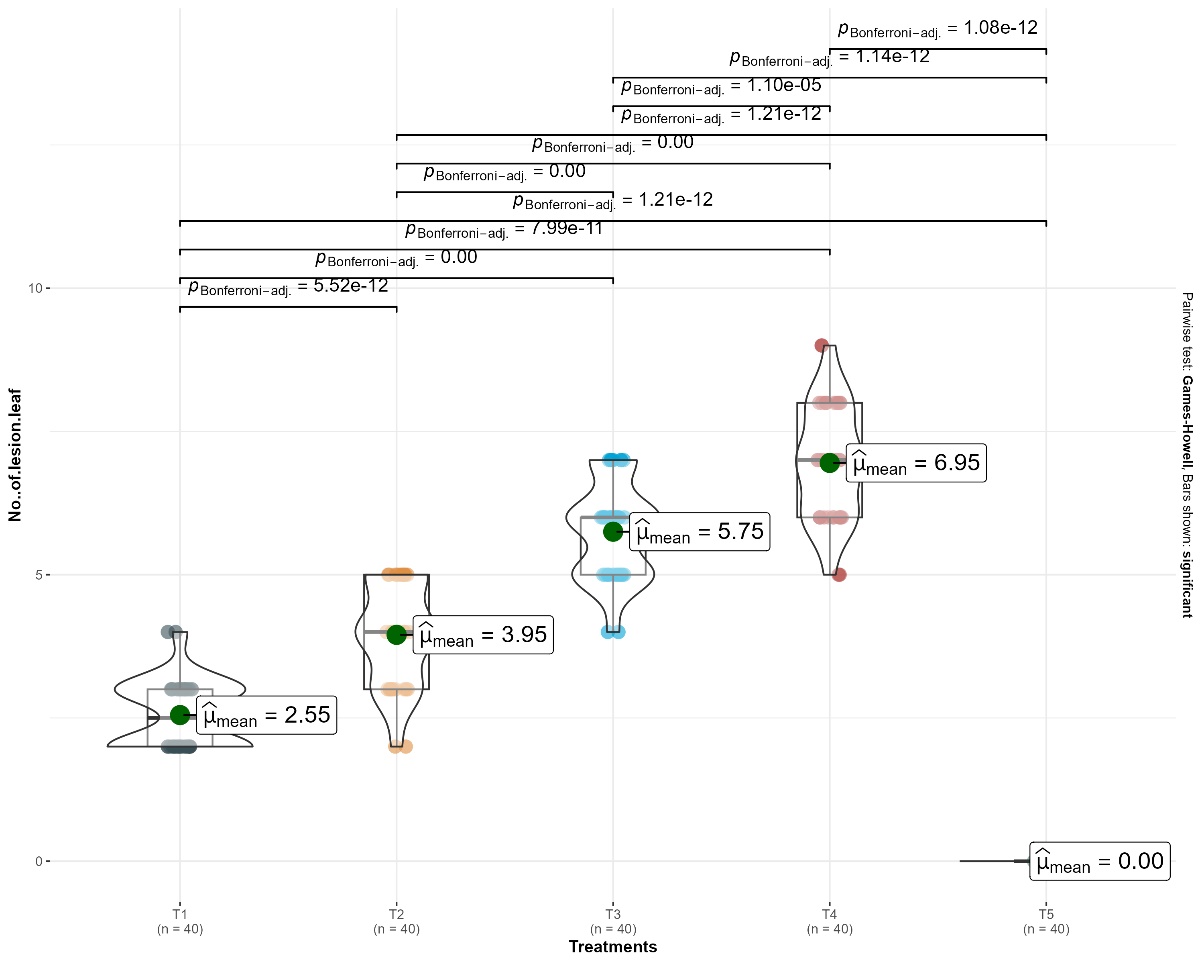


**FigureA1: Plots employing Games Howell post hoc test for elucidating significant differences among different groups for number of lesions per leaf [T1: Uninjured inoculated plants; T2: Mechanically injured inoculated plants; T3: *Thrips parvispinus* injured plants; T4: *Thrips tabaci* injured plant]**


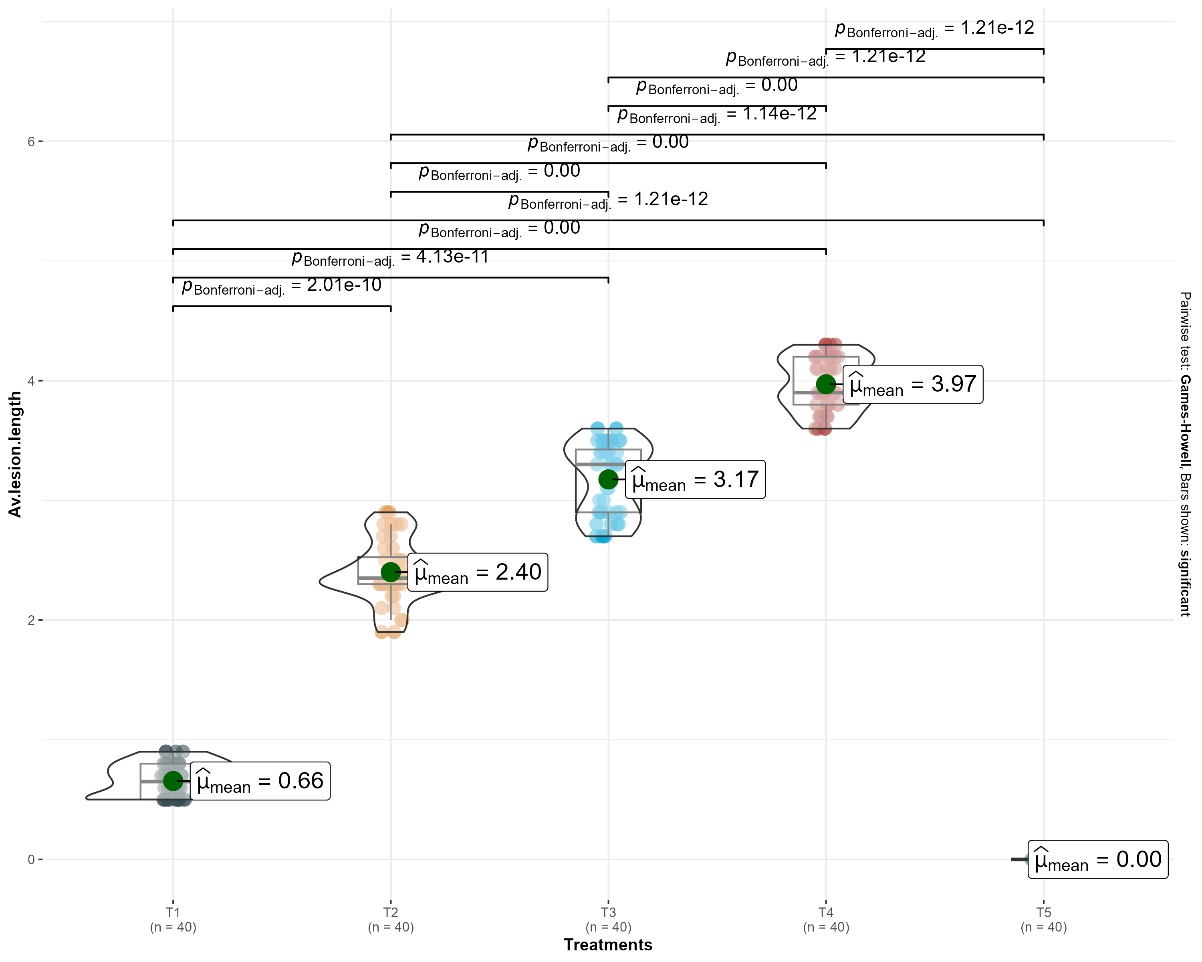


**Figure A2: Plots employing Games Howell post hoc test for elucidating significant differences among different groups for average lesions length [T1: Uninjured inoculated plants; T2: Mechanically injured inoculated plants; T3: *Thrips parvispinus* injured plants; T4: *Thrips tabaci* injured plant]**


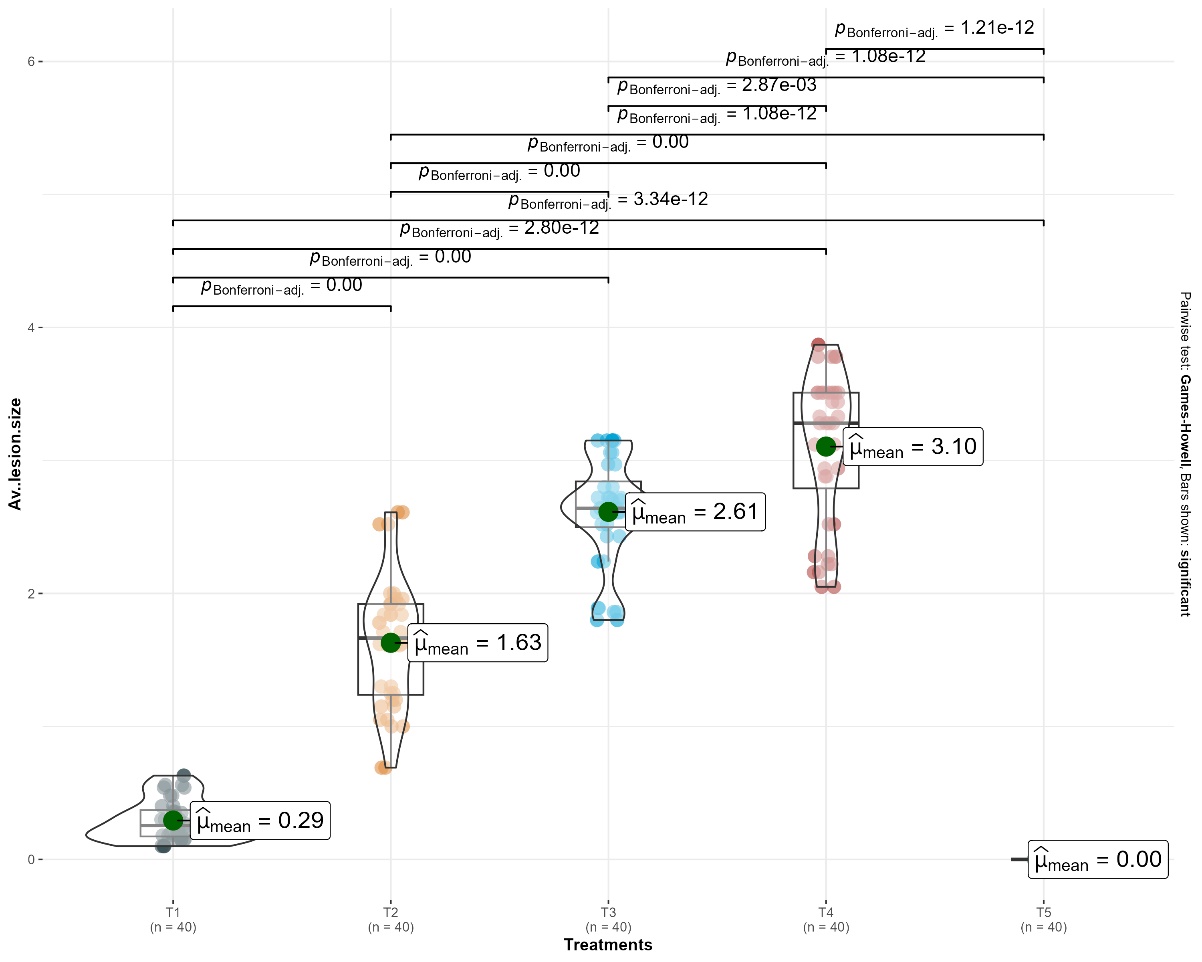


**Figure A3: Plots employing Games Howell post hoc test for elucidating significant differences among different groups for average lesion size [T1: Uninjured inoculated plants; T2: Mechanically injured inoculated plants; T3: *Thrips parvispinus* injured plants; T4: *Thrips tabaci* injured plant]**


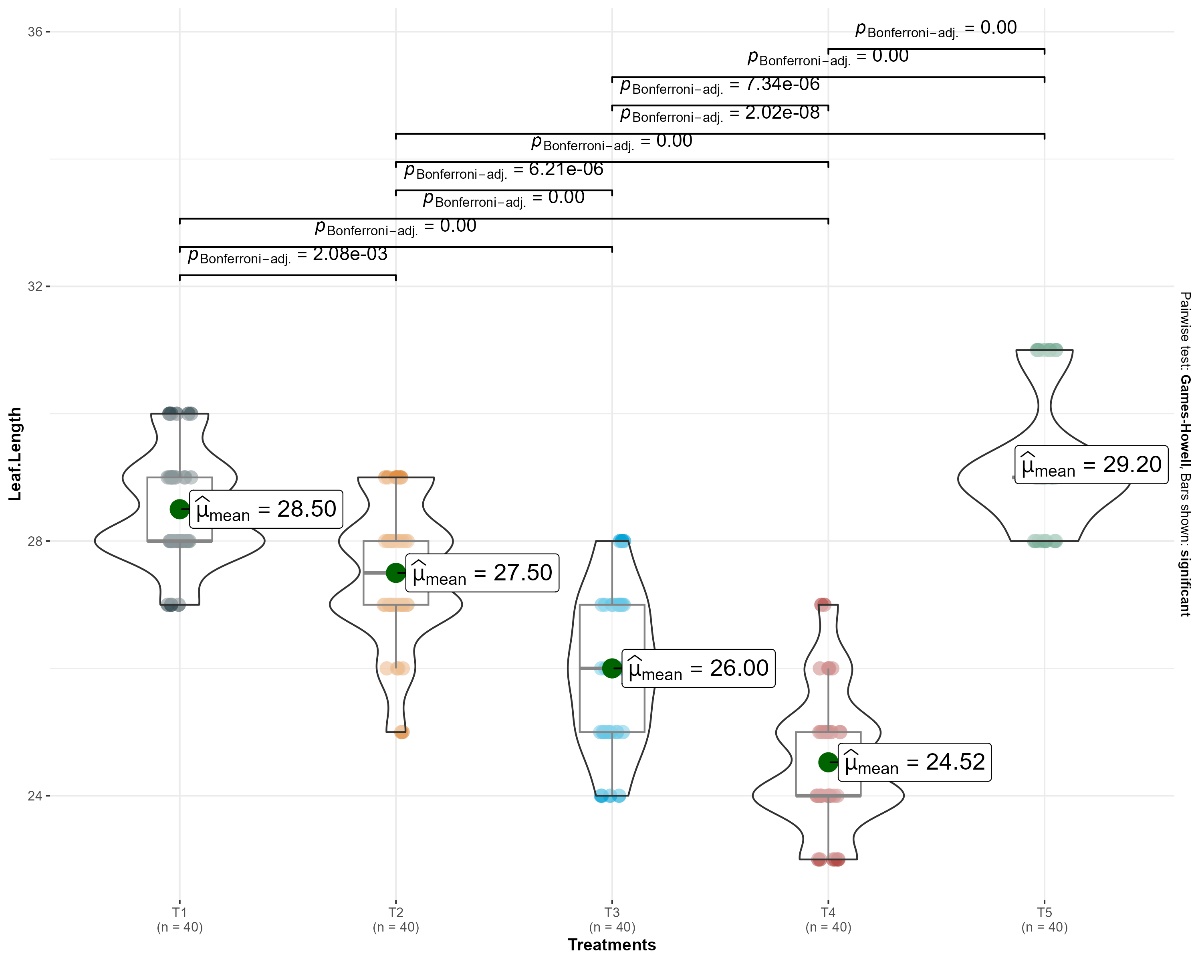


**Figure A4: Plots employing Games Howell post hoc test for elucidating significant differences among different groups for leaf length [T1: Uninjured inoculated plants; T2: Mechanically injured inoculated plants; T3: *Thrips parvispinus* injured plants; T4: *Thrips tabaci* injured plant]**


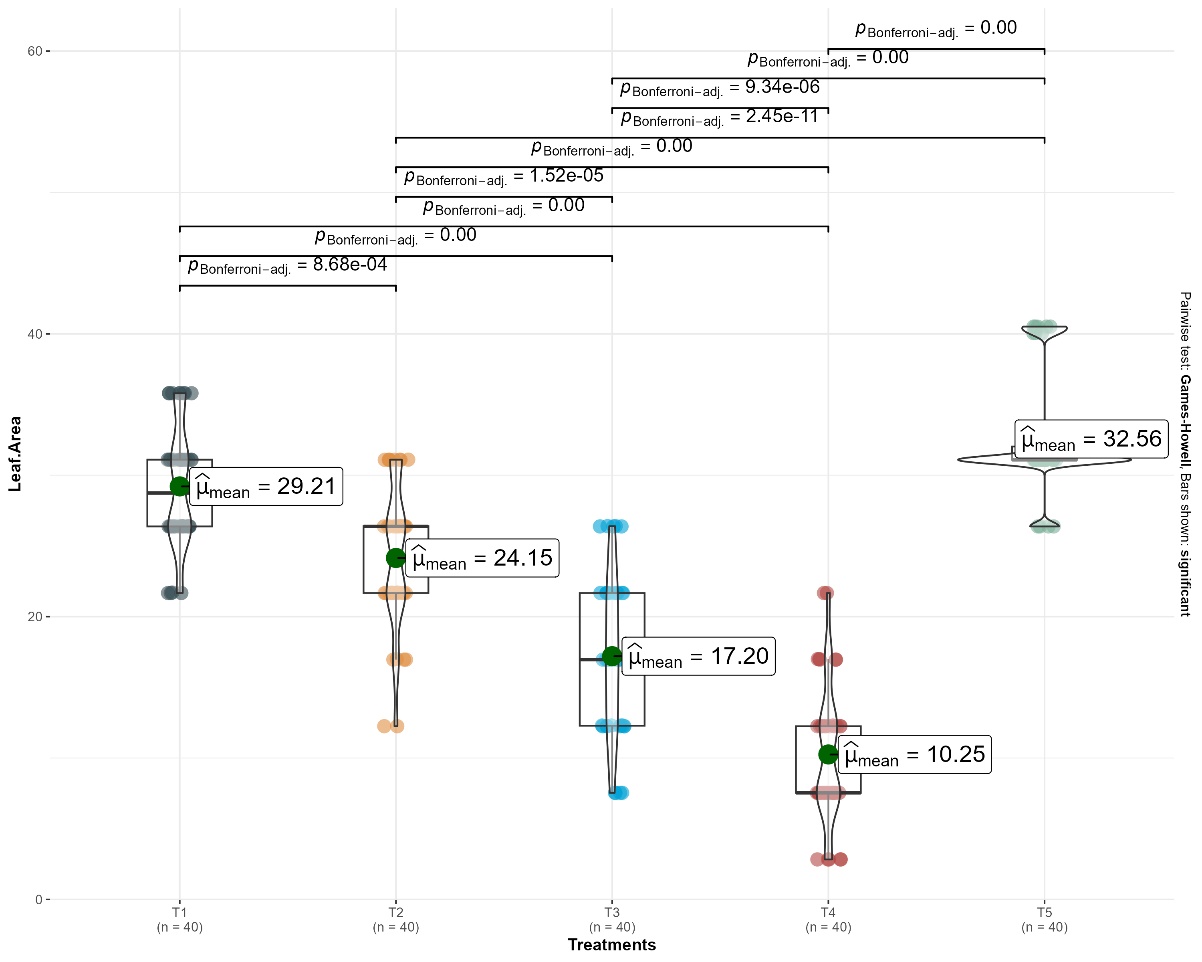


**Figure A5: Plots employing Games Howell post hoc test for elucidating significant differences among different groups for leaf area [T1: Uninjured inoculated plants; T2: Mechanically injured inoculated plants; T3: *Thrips parvispinus* injured plants; T4: *Thrips tabaci* injured plant]**


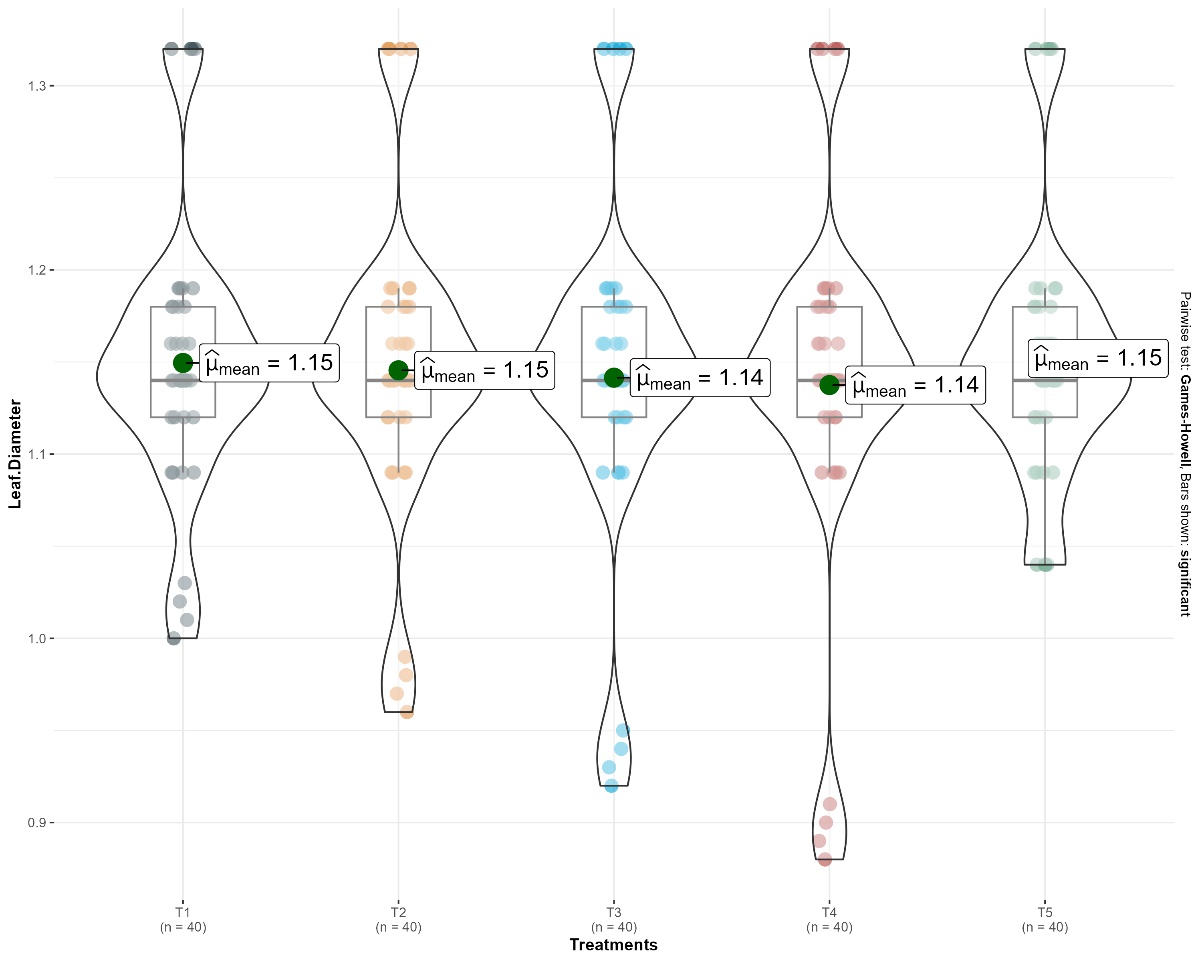


**Figure A6: Plots employing Games Howell post hoc test for elucidating significant differences among different groups for leaf diameter [T1: Uninjured inoculated plants; T2: Mechanically injured inoculated plants; T3: *Thrips parvispinus* injured plants; T4: *Thrips tabaci* injured plant]**


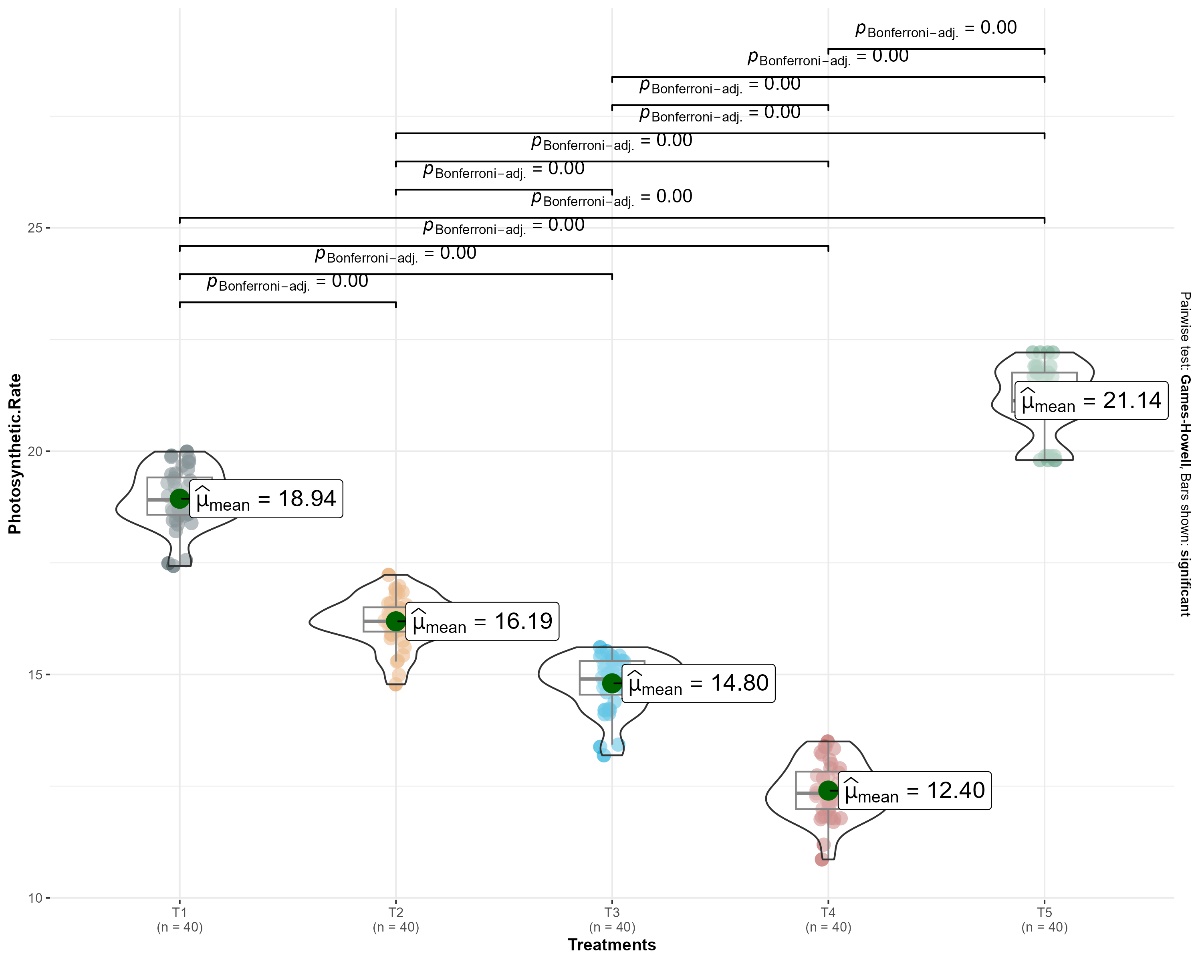


**Figure A7: Plots employing Games Howell post hoc test for elucidating significant differences among different groups for photosynthetic rate [T1: Uninjured inoculated plants; T2: Mechanically injured inoculated plants; T3: *Thrips parvispinus* injured plants; T4: *Thrips tabaci* injured plant]**


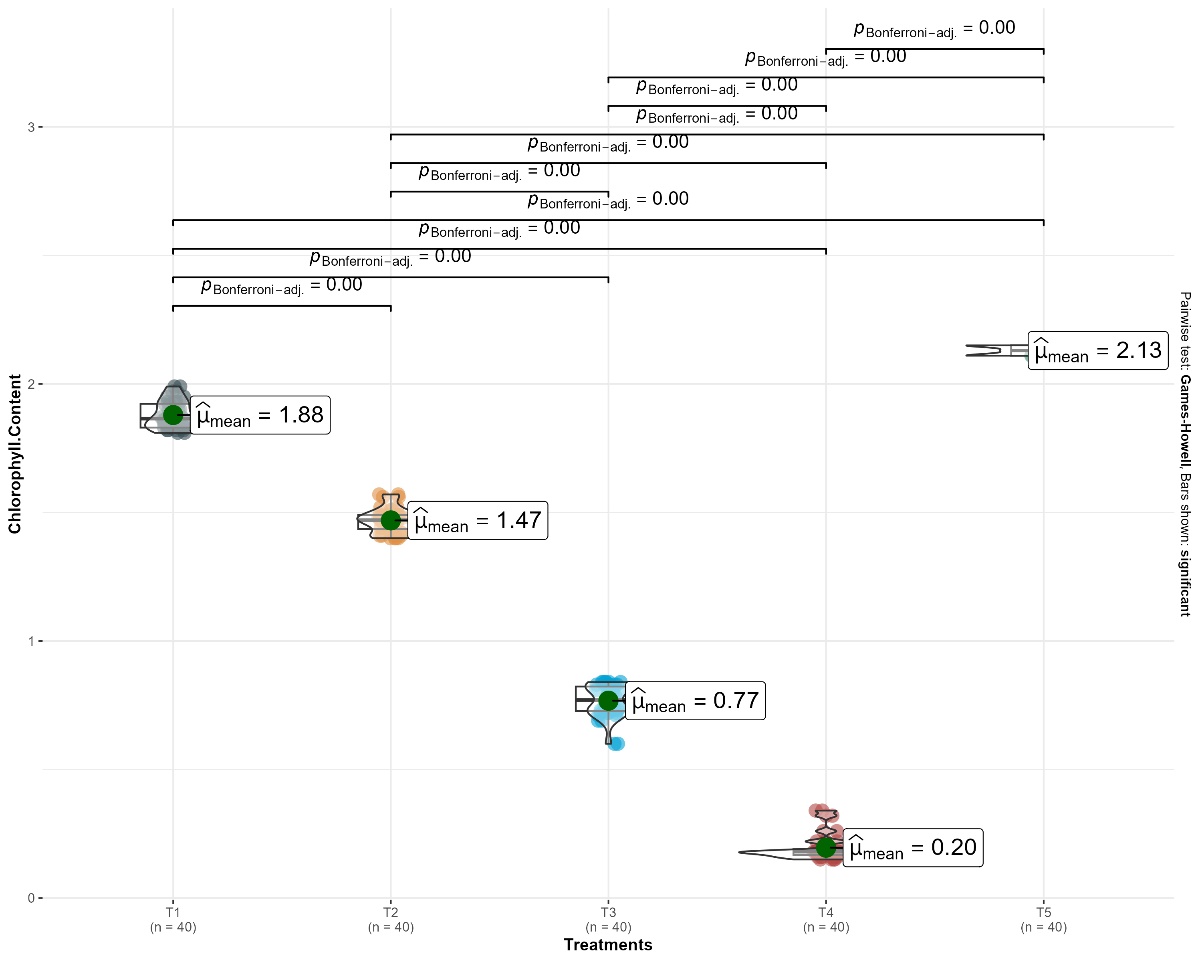


**Figure A8: Plots employing Games Howell post hoc test for elucidating significant differences among different groups for chlorophyll content [T1: Uninjured inoculated plants; T2: Mechanically injured inoculated plants; T3: *Thrips parvispinus* injured plants; T4: *Thrips tabaci* injured plant]**


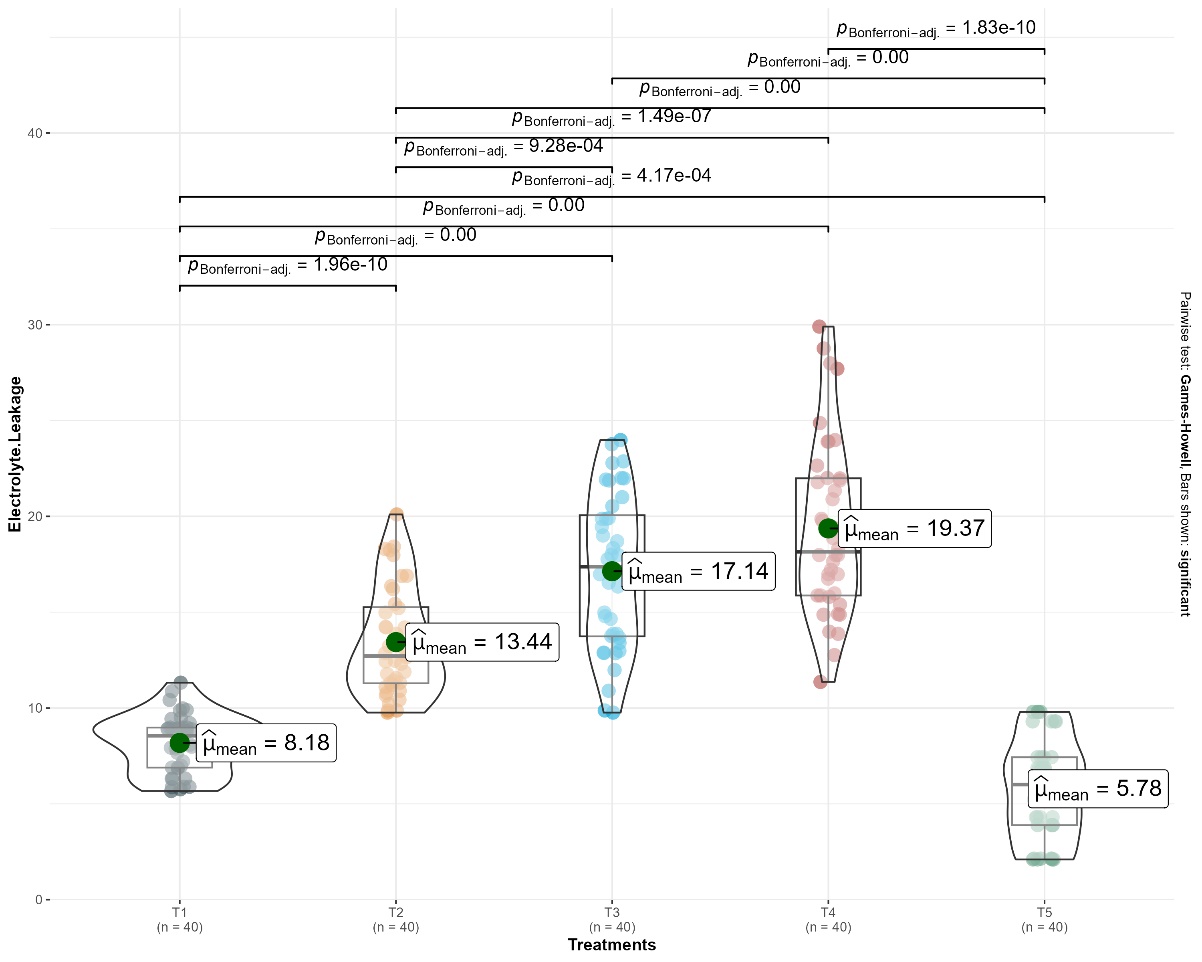


**Figure A9: Plots employing Games Howell post hoc test for elucidating significant differences among different groups for electrolyte leakage [T1: Uninjured inoculated plants; T2: Mechanically injured inoculated plants; T3: *Thrips parvispinus* injured plants; T4: *Thrips tabaci* injured plant]**


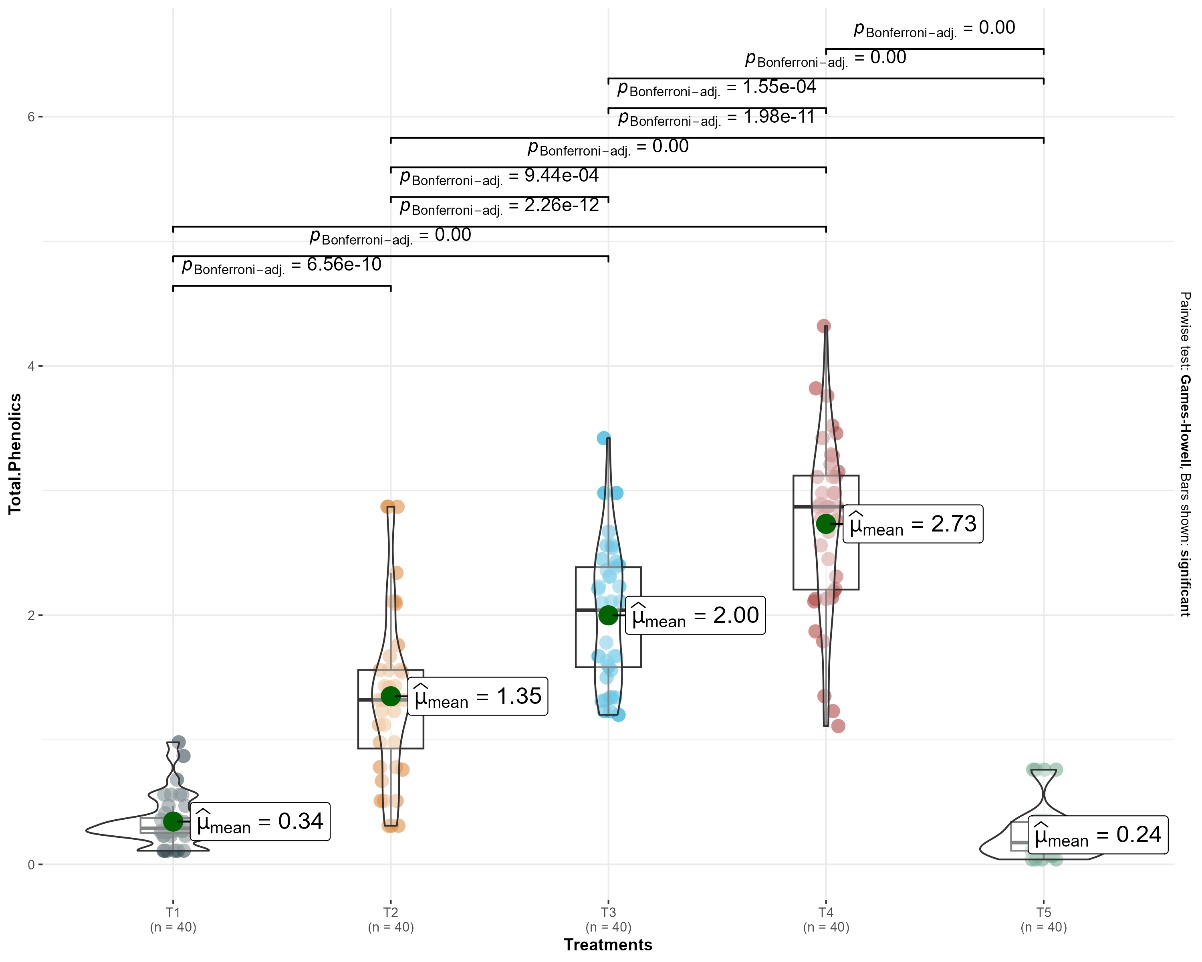


**Figure A10: Plots employing Games Howell post hoc test for elucidating significant differences among different groups for total phenolics [T1: Uninjured inoculated plants; T2: Mechanically injured inoculated plants; T3: *Thrips parvispinus* injured plants; T4: *Thrips tabaci* injured plant]**


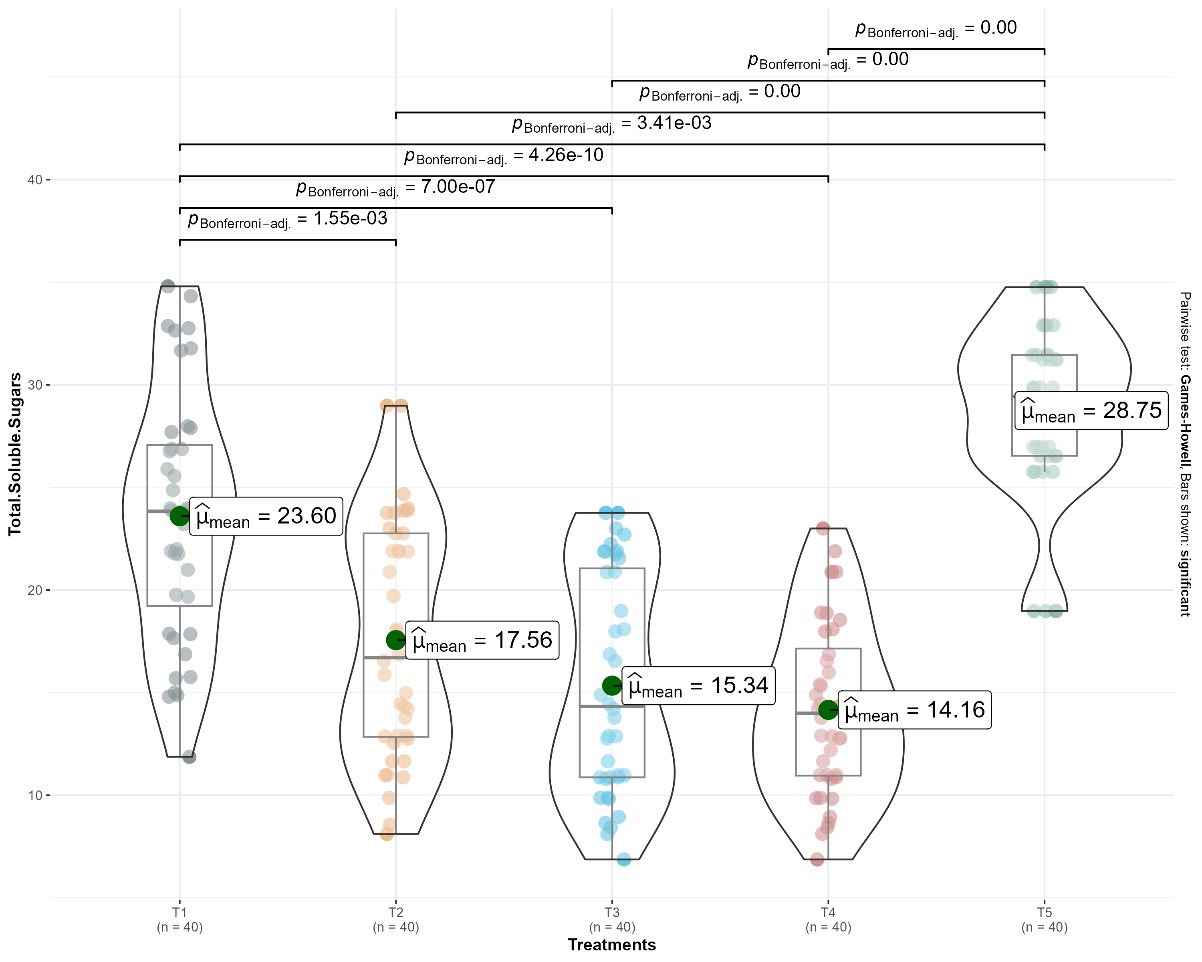


**Figure A11: Plots employing Games Howell post hoc test for elucidating significant differences among different groups for total soluble sugars [T1: Uninjured inoculated plants; T2: Mechanically injured inoculated plants; T3: *Thrips parvispinus* injured plants; T4: *Thrips tabaci* injured plant]**


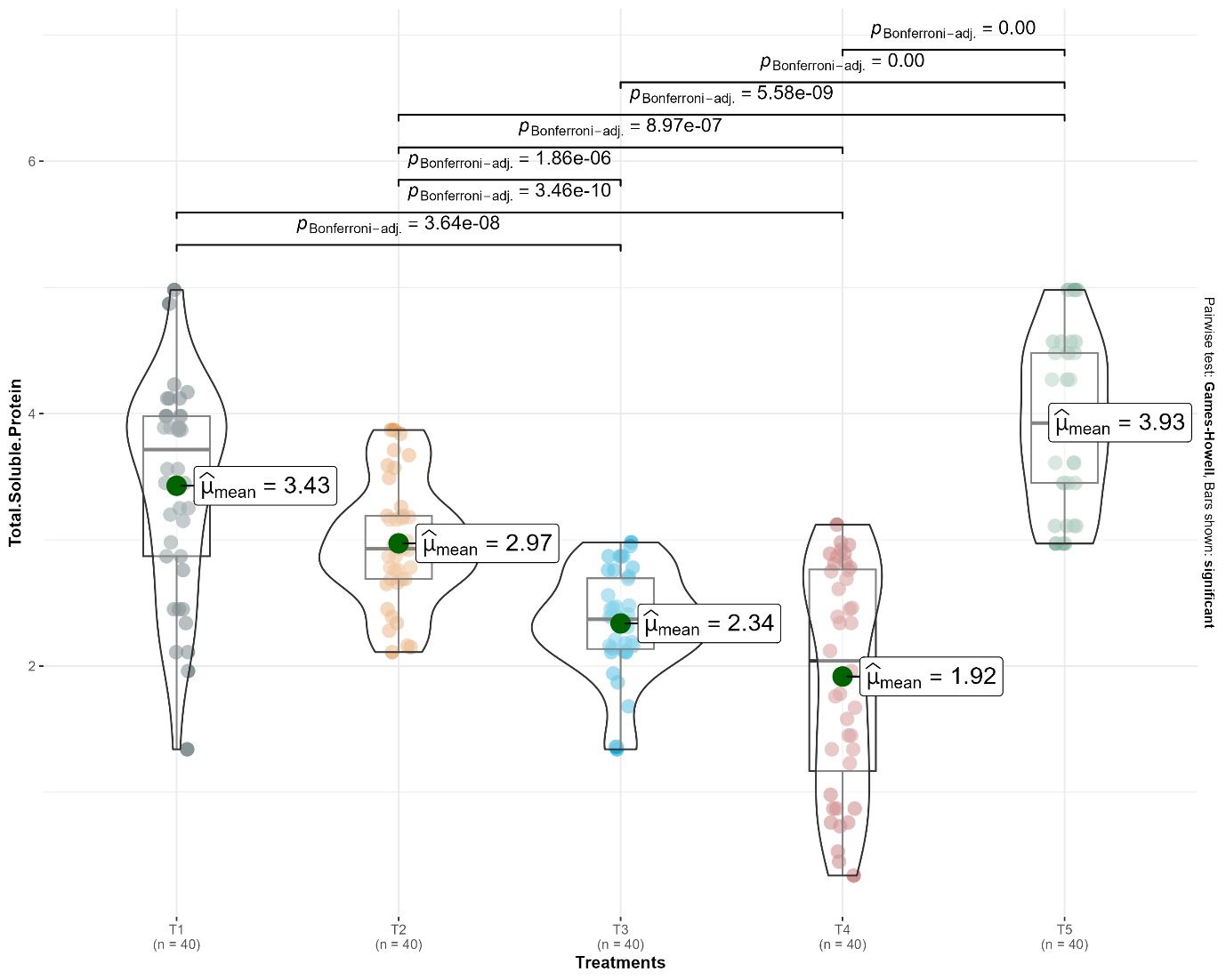


**Figure A12: Plots employing Games Howell post hoc test for elucidating significant differences among different groups for total soluble proteins [T1: Uninjured inoculated plants; T2: Mechanically injured inoculated plants; T3: *Thrips parvispinus* injured plants; T4: *Thrips tabaci* injured plant]**


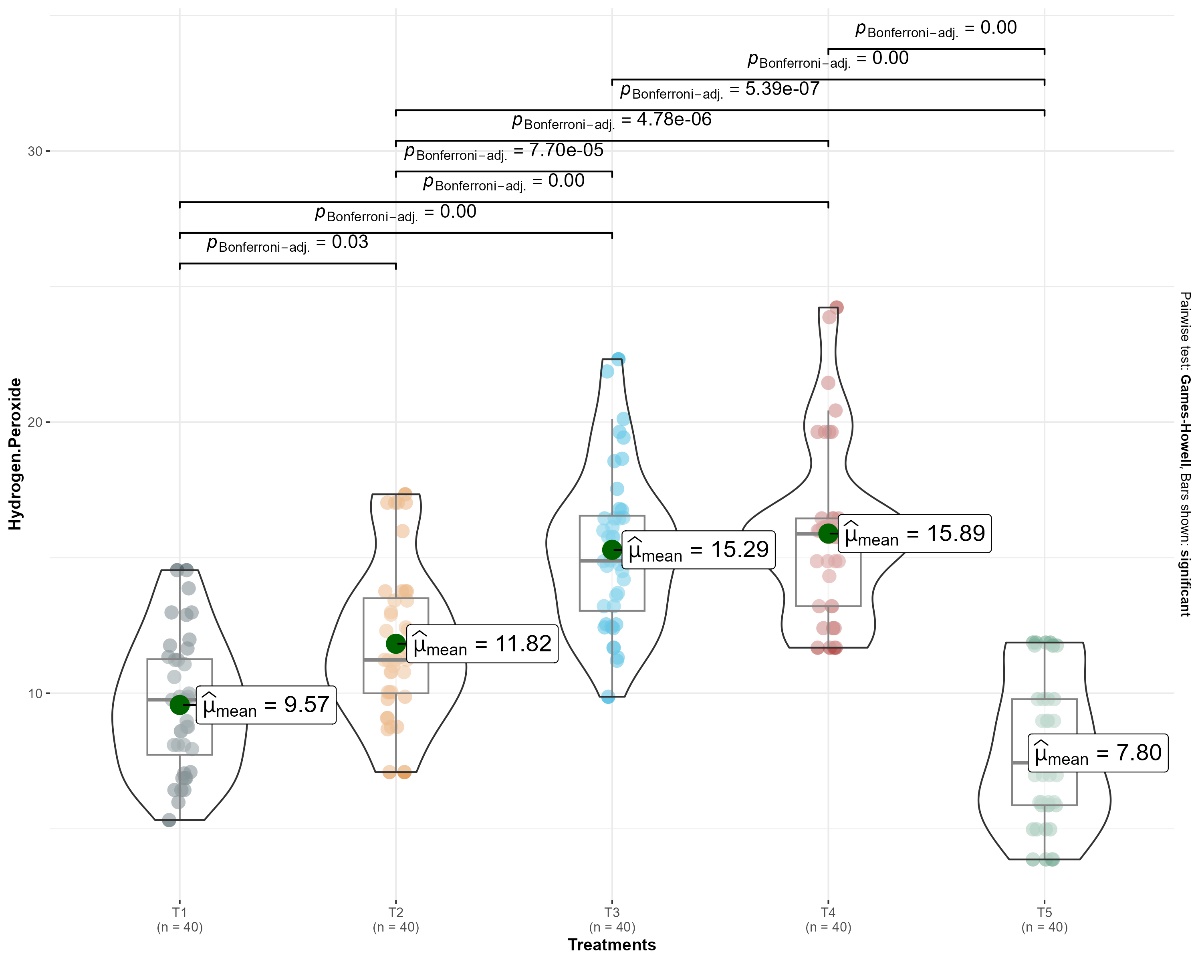


**Figure A13: Plots employing Games Howell post hoc test for elucidating significant differences among different groups for hydrogen peroxide [T1: Uninjured inoculated plants; T2: Mechanically injured inoculated plants; T3: *Thrips parvispinus* injured plants; T4: *Thrips tabaci* injured plant]**
